# Dietary long-chain fatty acids promote colitis by regulating palmitoylation of STAT3 through CD36-mediated endocytosis

**DOI:** 10.1038/s41419-024-06456-5

**Published:** 2024-01-17

**Authors:** Yuping Wei, Jinting Li, Jiao Li, Chuan Liu, Xingzhou Guo, Zhengru Liu, Luyun Zhang, Shenglan Bao, Xiaohan Wu, Wenhao Su, Xiaoli Wang, Jixiang Zhang, Weiguo Dong

**Affiliations:** 1 Department of Gastroenterology, Renmin Hospital of Wuhan University, Wuhan, Hubei Province China; 2 Key Laboratory of Hubei Province for Digestive System Disease, Wuhan, Hubei Province China; 3 Central Laboratory, Renmin Hospital of Wuhan University, Wuhan, Hubei Province China; 4 Department of Gastroenterology, The First Affiliated Hospital of Kunming Medical University, Kunming, China; 5 Department of Gastroenterology, Shengjing Hospital of China Medical University, Shenyang, China; 6 Department of Plastic Surgery, Renmin hospital of Wuhan University, Wuhan, Hubei Province China

**Keywords:** Ulcerative colitis, Medical research

## Abstract

The Western diet, characterized by its high content of long-chain fatty acids (LCFAs), is widely recognized as a significant triggering factor for inflammatory bowel disease (IBD). While the link between a high-fat diet and colitis has been observed, the specific effects and mechanisms remain incompletely understood. Our study provides evidence that the diet rich in LCFAs can disrupt the integrity of the intestinal barrier and exacerbate experimental colitis in mice. Mechanistically, LCFAs upregulate the signal transducer and activator of transcription-3 (STAT3) pathway in the inflammatory model, and STAT3 knockout effectively counters the pro-inflammatory effects of LCFAs on colitis. Specifically, palmitic acid (PA), a representative LCFA, enters intestinal epithelial cells via the cluster of differentiation 36 (CD36) pathway and participates in the palmitoylation cycle of STAT3. Inhibiting this cycle using pharmacological inhibitors like 2-Bromopalmitate (2-BP) and ML349, as well as DHHC7 knockdown, has the ability to alleviate inflammation induced by PA. These findings highlight the significant role of dietary LCFAs, especially PA, in the development and progression of IBD. Diet adjustments and targeted modulation offer potential therapeutic strategies for managing this condition.

**Figure:**
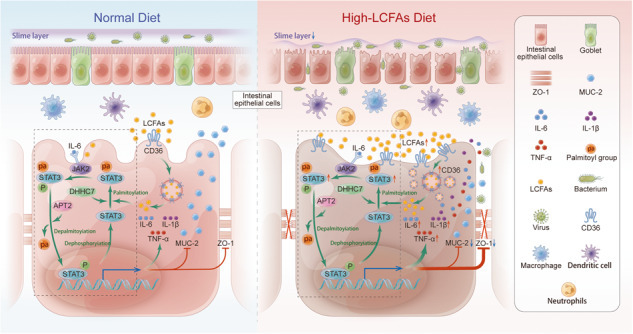
Model of LCFAs involvement in the palmitoylation cycle of STAT3 upon internalization into cells. Following cellular uptake through CD36, LCFAs are converted to palmitoyl-CoA. In the presence of DHHC7, palmitoyl-CoA binds to STAT3 at the C108 site, forming palmitoylated STAT3. Palmitoylation further promotes phosphorylation at the Y705 site of STAT3. Subsequently, palmitoylated STAT3 undergoes depalmitoylation by APT2 and translocates to the nucleus to exert its biological functions.

Model of LCFAs involvement in the palmitoylation cycle of STAT3 upon internalization into cells. Following cellular uptake through CD36, LCFAs are converted to palmitoyl-CoA. In the presence of DHHC7, palmitoyl-CoA binds to STAT3 at the C108 site, forming palmitoylated STAT3. Palmitoylation further promotes phosphorylation at the Y705 site of STAT3. Subsequently, palmitoylated STAT3 undergoes depalmitoylation by APT2 and translocates to the nucleus to exert its biological functions.

## Introduction

Inflammatory bowel disease (IBD), comprising Crohn’s disease and ulcerative colitis (UC), is a complex idiopathic intestinal inflammatory condition that primarily affects the ileum, colon, and rectum. Its pathogenesis involves a combination of genetic, environmental, intestinal barrier, and immune response factors [1]. Previous investigations have indicated that the adoption of a Westernized diet increases susceptibility to IBD. Western diets are characterized by an elevated intake of simple carbohydrates and long-chain fatty acids (LCFAs), alongside reduced fiber consumption. LCFAs constitute 95% of triglycerides in these diets [2]. High-fat diets (HFD) have been implicated in the exacerbation of colitis by promoting oxidative stress and mitochondrial dysfunction [3] and by perturbing mucosal dendritic cell homeostasis [4]. However, the specific mechanisms underlying these effects require further elucidation.

LCFAs typically refer to fatty acids (FAs) with 16 or more carbon atoms and can be categorized as saturated or unsaturated based on the presence of double bonds in the carbon chain [5]. Palmitic acid (PA C16:0) and stearic acid (SA C18:0) are common saturated LCFAs found abundantly in various foods, such as palm oil, coconut oil, cocoa butter, dairy products, and meat [6–8]. PA, as the most prevalent saturated FA in the human body, plays vital roles in cellular membrane composition, secretory lipid production, and transport [6–9].

Recent research has increasingly links the signal transducer and activator of transcription-3 (STAT3) pathway to IBD [10, 11], with the IL-6/STAT3 signaling pathway consistently recognized as a noteworthy factor in colitis development [12, 13]. Earlier research suggests that STAT3 can undergo S-palmitoylation through DHHC7 catalysis, and acyl-protein thioesterase-2 (APT2; also known as lysophospholipase 2, LYPLA2) is primarily responsible for depalmitoylation, forming what is known as the palmitoylation cycle of STAT3. This palmitoylation cycle of STAT3 is thought to play a notable role in the initiation and progression of colitis [14].

Cluster of differentiation 36 (CD36), a multifunctional membrane glycoprotein, functions as an FA transporter, facilitating the cellular uptake of LCFAs [15, 16]. Previous studies have indicated that FAs or HFD can promote CD36 transcription and upregulation of its functions [17]. Knockdown of CD36 in mice and cells has been shown to reduce HFD and FA-induced fatty liver, insulin resistance [18], and cancer metastasis [19]. However, there is currently a lack of evidence regarding the role of CD36 in IBD.

Our investigation has indicated that dietary LCFAs can enter intestinal epithelial cells via CD36-mediated uptake and endocytosis, thereby participating in the palmitoylation cycle of STAT3 and exacerbating colonic inflammation, which plays a notable role in the development of UC.

## Results

### FFAs, especially LCFAs, are upregulated in the serum of patients with UC

**Fig. 1:**
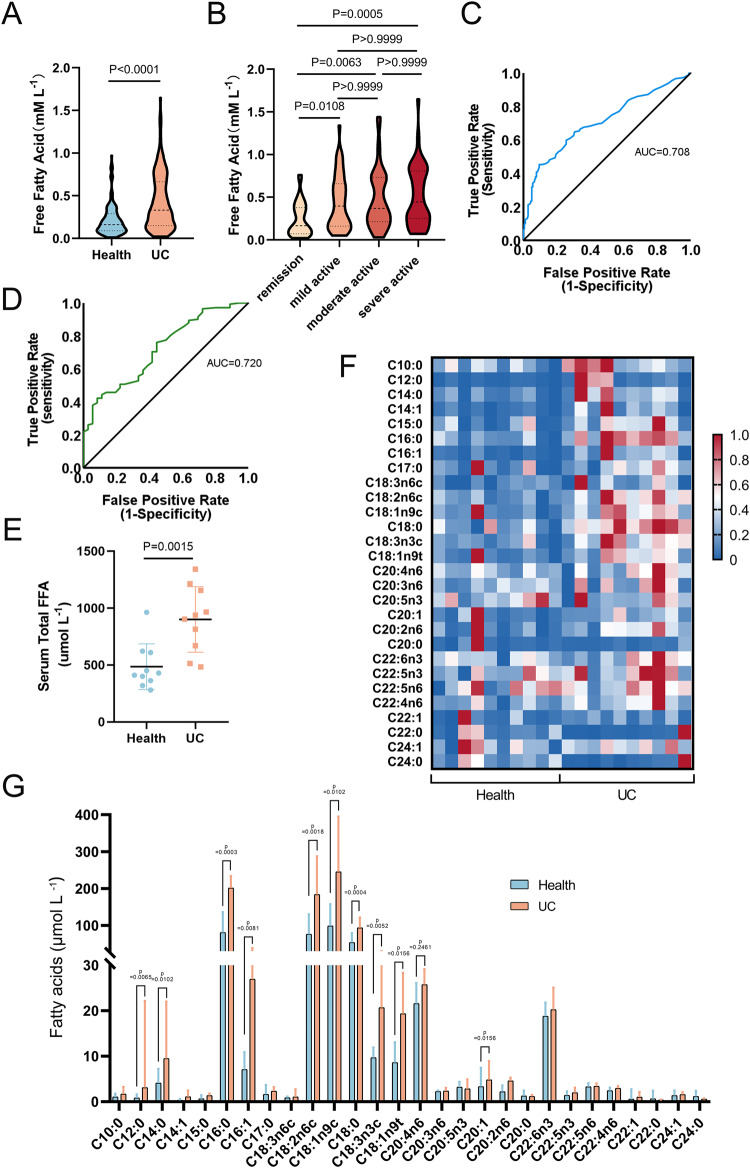
FFAs, especially LCFA, are upregulated in the serum of patients with UC. **A** Serum FFA concentrations were compared between UC patients and healthy controls (*n* = 178). **B** Serum FFA concentrations were compared among UC patients in remission, and those with mild, moderate, and severe disease activity. **C** ROC curve analysis was performed to assess the predictive value of FFA for UC. **D** ROC curve analysis was performed to assess the predictive value of FFA for active UC. **E** Total FFA concentrations in serum were measured by targeted GC-FID/MS analysis in UC patients and healthy controls (*n* = 10). **F** Heatmap analysis to show the normalized FA concentration of each sample. **G** Histograms were plotted based on the FFA concentrations for each sample in the two groups, with blue bars representing healthy controls and orange bars representing UC patients. *P* values were determined using unpaired two-tailed Student’s t-test (**E**, **G**), unpaired two-tailed Mann-Whitney test (**A**), or Kruskal-Wallis test (**B**). ***p < 0.001; **p < 0.01; *p < 0.05.

This study included 178 discharged patients with a confirmed diagnosis of UC and 178 individuals who had been receiving other health examinations as the control group. Baseline characteristics showed no statistically significant differences in sex distribution and age between the UC and control groups (Table S1). However, the UC group exhibited higher levels of serum-free fatty acids (FFAs) compared to the control group (*P* < 0.0001) (Fig. 1A).

Among the UC patients, those in remission (*n* = 36) had lower serum FFA levels compared to those in the mild (*n* = 52) (*P* = 0.0108), moderate (*n* = 42) (*P* = 0.0063), and severe (*n* = 48) (*P* = 0.0005) active phases. No significant differences in FFA levels were observed among varying degrees of activity within the active phase (Fig. 1B). FFA showed potential as a biomarker for distinguishing UC patients from healthy controls (AUC = 0.708) (Fig. 1C), and for discriminating between remission and active phases (AUC = 0.720) (Fig. 1D) based on receiver operating characteristics (ROC) curve analysis.

For further analysis, 10 cases from both the UC and control groups were collected for Targeted Gas Chromatography–Flame Ionization Detector/Mass Spectrometry (GC-FID/MS) analysis. The UC group had higher total serum FFA levels than the control group (*P* = 0.0015) (Fig. 1E). Overall, the levels of FAs were more abundant in the UC group compared to the healthy control group, particularly the elevated levels of LCFAs with carbon chain lengths of 16, 18, and 22, as shown in the heatmap (Fig. 1F).

The concentrations of various FAs were analyzed (Table S2) and depicted using histograms (Fig. 1G). Among the 28 detectable FAs, C16:0, C18:0, C18:2n6c, and C18:1n9C were the most abundant, and statistically significant differences were observed in their concentrations between the UC and control groups. Notably, the most prominent disparities were found for C16:0 (*P* = 0.0003) and C18:0 (*P* = 0.0004), corresponding to PA and SA, respectively.

In summary, our investigation underscores a substantial upregulation of FFAs, especially LCFAs, in the serum of UC patients, particularly those in the active phase. PA and SA demonstrated the most profound disparities in abundance.

### A high-LCFA diet exacerbates dextran sulfate sodium (DSS)-induced colitis of wild-type (WT) mice

**Fig. 2:**
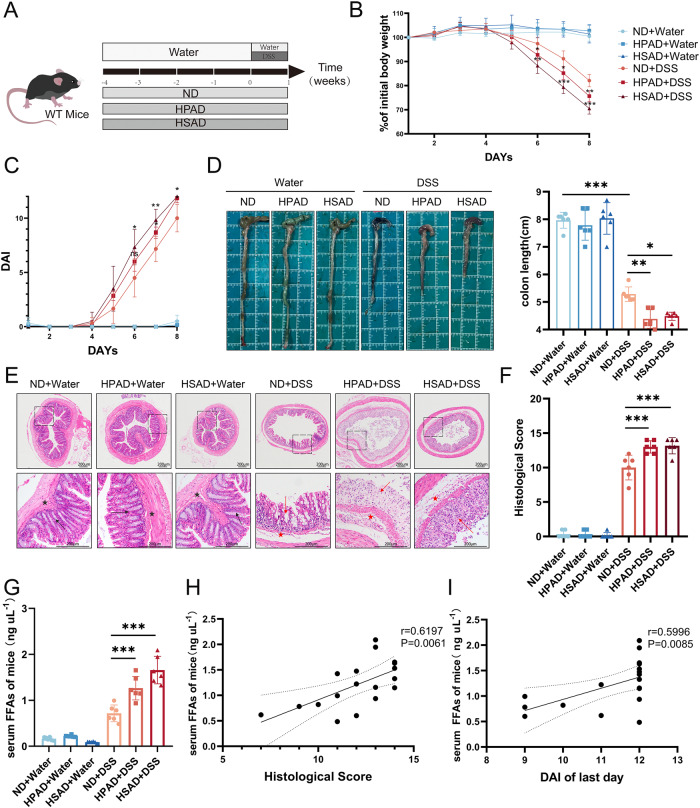
A High-LCFA diet exacerbates DSS-induced colitis of WT mice. **A** WT Mice (*n* = 6) were given ND, HPAD, and HSAD for 4 weeks, followed by continuous administration of 3% DSS in drinking water for 7 days in the colitis groups. **B** Changes in mice body weight. **C** Changes in DAI; **D** Representative colon morphology and length with quantification in mice; **E** Representative images of colon H&E staining. Well-preserved mucosal crypts and villi structure (black arrow), and healthy submucosa with no inflammation (black star); Mucosal erosions, ulceration, and mucosal atrophy (red arrows), and swollen submucosa with inflammatory cell infiltration (red star). **F** Histological scoring of mice based on colon section morphology; **G** Comparison of levels of FFAs in mice serum. **H** Spearman correlation analysis between levels of serum FFAs and pathological histological scoring in mice from the ND + DSS group, HPAD + DSS group, and HSAD + DSS group. **I** Spearman correlation analysis between levels of serum FFAs and DAI of last day from the ND + DSS group, HPAD + DSS group, and HSAD + DSS group. The data were presented as mean ± SD. *P* values were determined using one-way or two-way ANOVA test.; ***p < 0.001, **p < 0.01, *p < 0.05.

The pro-inflammatory potency of a high-LCFA diet was evaluated in the DSS-induced colitis model (Fig. 2A). Our study revealed that mice treated with the high PA diet (HPAD) or the high SA diet (HSAD) exhibited more pronounced weight loss (Fig. 2B), a higher Disease Activity Index (DAI) (Fig. 2C), reduced colon length (Fig. 2D), and increased histological scores (Fig. 2F) compared to mice treated with normal diet (ND) in the colitis model. Mice administered HPAD or HSAD displayed severe colon inflammation, characterized by inflammatory cell infiltration, epithelial defects, and crypt atrophy (Fig. 2E). Furthermore, administration of HPAD or HSAD alone had minimal effects on the mice (Fig. 2B–F).

We measured the serum FFA levels of mice and observed that they were elevated only in the colitis mouse models, and the increase in HPAD or HSAD-treated mice was more significant compared to ND-treated mice (Fig. 2G). Spearman’s correlation analysis revealed a positive correlation between serum FFA levels and both the histological scores (r = 0.619, *P* = 0.0061) (Fig. 2H) and the DAI (r = 0.5996, *P* = 0.0085) (Fig. 2I) of the mice on the final day of the experiment.

In summary, a high-LCFA diet exacerbates DSS-induced colitis in WT mice, as evidenced by increased inflammation and severity of colitis symptoms.

### Dietary LCFAs exacerbate intestinal barrier damage in DSS-induced colitis mice

**Fig. 3:**
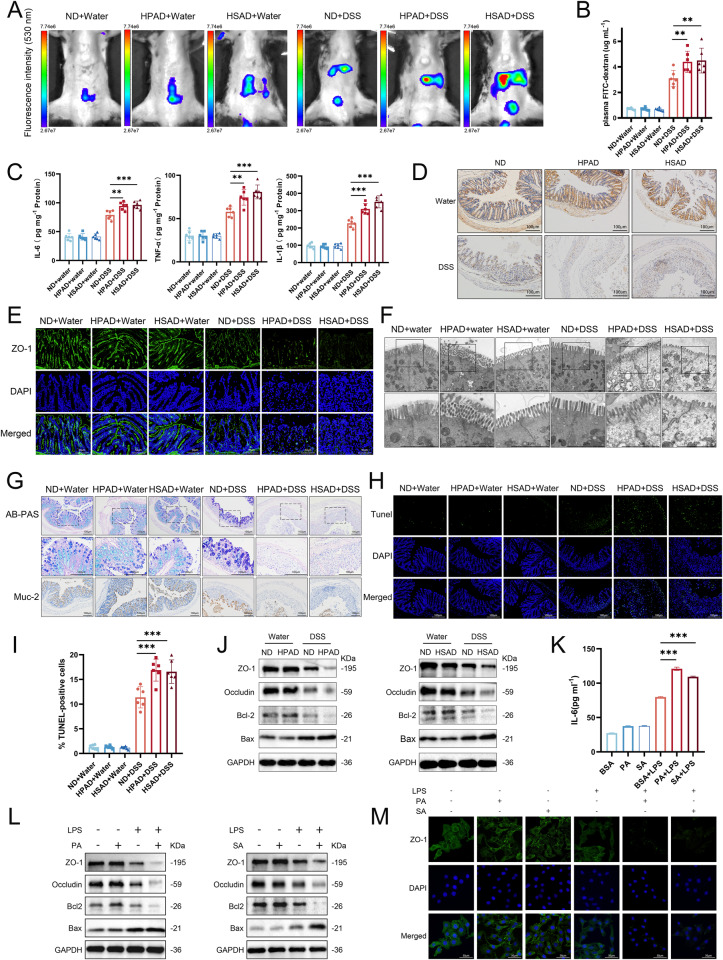
High-LCFAs diet exacerbates intestinal barrier damage in DSS-induced colitis mice. **A** The remaining fluorescence and absorption of FITC-dextran. Red represents the highest intensity, while dark purple indicates the lowest intensity; **B** The plasmatic concentration of FITC-dextran (*n* = 6); **C** Levels of IL-6, TNF-α, and IL-1β in colonic tissues of mice (*n* = 6); **D** Representative images of colonic tissue IHC staining for ZO-1; **E** Representative images of colonic tissue IF staining for ZO-1; **F** Scanning electron microscopy images of mouse colonic tissue sections, with magnified regions showing tight junctions; **G** Representative images of colonic tissue staining with AB-PAS and IHC of MUC-2; **H** TUNEL staining of colonic tissue; **I** Statistical analysis of TUNEL-positive cells; **J** Western blotting of colonic tissues. In vitro experiments: NCM460 cells stimulated with 1ug mL^−1^ LPS in combination with 200 µM PA, 100 µM SA, or BSA for 24 h; **K** ELISA measurement of IL-6 levels in cell culture supernatants; **L** Western blotting of cells; **M** Representative images of IF staining of ZO-1 IF in NCM460 cell. Statistical significance was determined using one-way ANOVA test. ***p < 0.001, **p < 0.01, *p < 0.05.

To explore the impact of LCFAs on IBD severity in pathogenesis, we utilized fluorescein isothiocyanate-labeled dextran (FITC-dextran) to assess intestinal barrier permeability in mice. The results revealed that both HPAD and HSAD exacerbated the absorption of FITC-Dextran in the liver and spleen of mice with DSS-induced colitis (Fig. 3A). Additionally, these diets increased the concentration of FITC-Dextran in the plasma four hours after gavage (Fig. 3B), indicating that LCFAs in the diet heightened intestinal permeability under pathological conditions.

Then we examined inflammatory cytokines and intestinal barrier markers in mouse intestines. In the colitis mice, the expression of IL-6, TNF-α, and IL-1β in the colon tissue was significantly increased after treatment with HPAD or HSAD (Fig. 3C).

The expression of zona occludens 1 (ZO-1) was downregulated in the inflammation groups and further decreased in mice treated with HPAD and HSAD compared to the ND group (Fig. 3D, E). Electron microscopy of the colonic epithelium showed that a high-LCFA diet aggravated changes in tight junction integrity in colitis (Fig. 3F). Additionally, the number of colonic epithelial goblet cells was reduced, and the production of mucus and the expression of mucin 2 (MUC-2) protein were inhibited in colitis mice treated with HPAD and HSAD. (Fig. 3G).

Apoptosis plays a role in causing dysfunction of the intestinal epithelial barrier and triggering inflammatory responses in DSS-induced colitis [20]. Both HPAD and HSAD treatment significantly increased the number of TUNEL-positive cells in colitis (Fig. 3H, I). Meanwhile, Western blotting indicated suppressed expression of ZO-1, occludin and anti-apoptotic protein BCL-2, along with increased expression of the pro-apoptotic protein Bax (Fig. 3J). Notably, no statistical differences were observed between control groups (Fig. 3A–J).

We observed a consistent trend in the expression of IL-6, ZO-1, occludin, Bcl-2, and Bax in the in vitro experiments (Fig. 3K–M).

In conclusion, these results indicated that LCFAs exacerbate intestinal barrier damage in mice with DSS-induced colitis.

### Dietary LCFAs upregulated the phosphorylation of STAT3

**Fig. 4:**
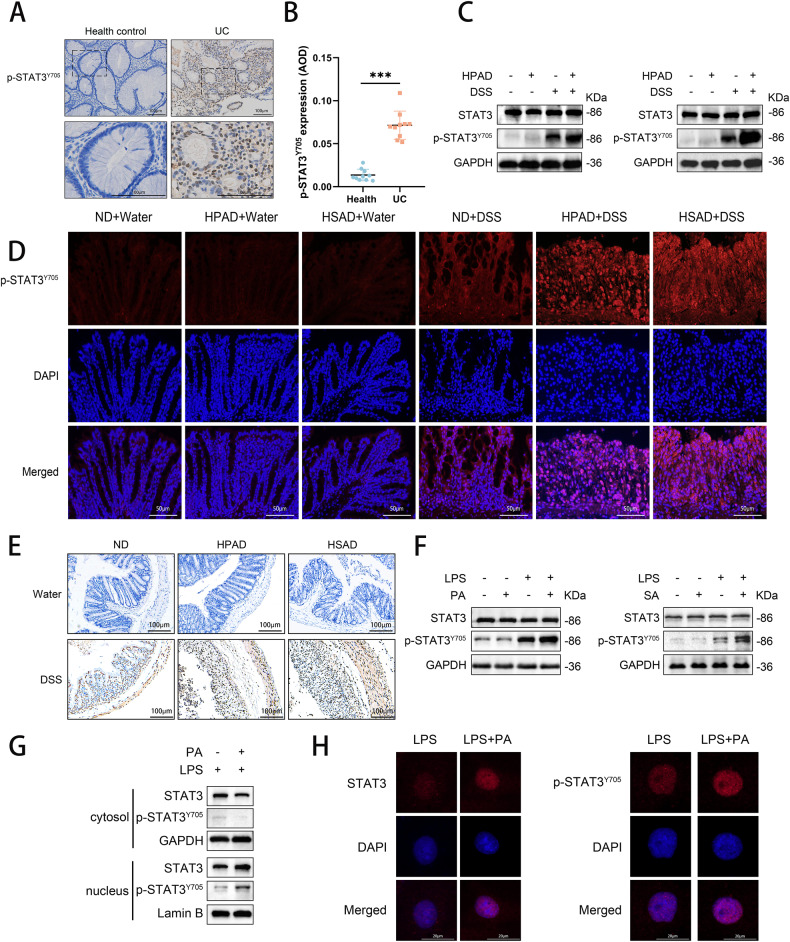
Dietary LCFAs upregulated the phosphorylation of STAT3 in vivo. **A** IHC staining of colonic specimens to assess the expression of p-STAT3^Y705^ in patients with UC compared to healthy controls. **B** Quantitative analysis of p-STAT3^Y705^ IHC staining in UC and healthy control groups. **C** Western blotting was conducted to determine the levels of STAT3 and p-STAT3^Y705^ in mice colonic tissues (Fig. 3). **D** IF staining of p-STAT3^Y705^ was performed on mouse colonic tissue sections. **E** IHC staining of p-STAT3^Y705^ was performed on mouse colonic tissue sections. NCM460 cells were stimulated with 1 μg mL^−1^ LPS in combination with 200 μM PA, 100 μM SA, or BSA alone as a control for 24 h. **F** Western blotting was performed to assess the levels of STAT3 and p-STAT3^Y705^ in the cells. **G** Western blotting to determine the levels of STAT3 and p-STAT3^Y705^ in the cytoplasm and nucleus of the cells. **H** IF staining of STAT3 and p-STAT3^Y705^. *P* values were determined by 2-tailed Student t test. ***p < 0.001, **p < 0.01, *p < 0.05.

Previous studies have revealed that PA can trigger STAT3 phosphorylation and exert biological effects in gastric cancer and prostate cancer models [21, 22]. In our study, we found that LCFAs increased IL-6 levels in the inflammatory model, prompting us to further explore the impact of LCFAs on the IL-6/STAT3 pathway in UC. Our findings revealed an upregulation of p-STAT3^Y705^ expression in UC patients compared to the healthy control group (Fig. 4A, B).

In mice, Western blotting revealed that administration of HPAD or HSAD in colitis-induced mice led to an increase in p-STAT3^Y705^ expression, while the total STAT3 level remained unchanged (Fig. 4C). Immunohistochemistry (IHC) and immunofluorescence (IF) analyses further validated these findings, showing increased p-STAT3 expression (Fig. 4D, E). These results led to the hypothesis that LCFAs exacerbate DSS-induced colitis by activating STAT3 phosphorylation. In vitro investigations also yielded consistent outcomes (Fig. 4F).

Furthermore, we evaluated the levels of STAT3 and p-STAT3 in nuclear and cytoplasmic proteins. Upon PA treatment in the LPS-induced inflammation model, we observed an upregulation of p-STAT3 expression primarily in nuclear proteins, while total STAT3 expression was upregulated in nuclear proteins but downregulated in cytosol proteins (Fig. 4G). IF analysis supported these findings (Fig. 4H).

Collectively, these results highlight that LCFAs aggravate colitis by enhancing STAT3 phosphorylation and its translocation into the nucleus.

### Suppression of STAT3 alleviates LCFA-induced exacerbated inflammation and intestinal barrier damage in experimental colitis

**Fig. 5:**
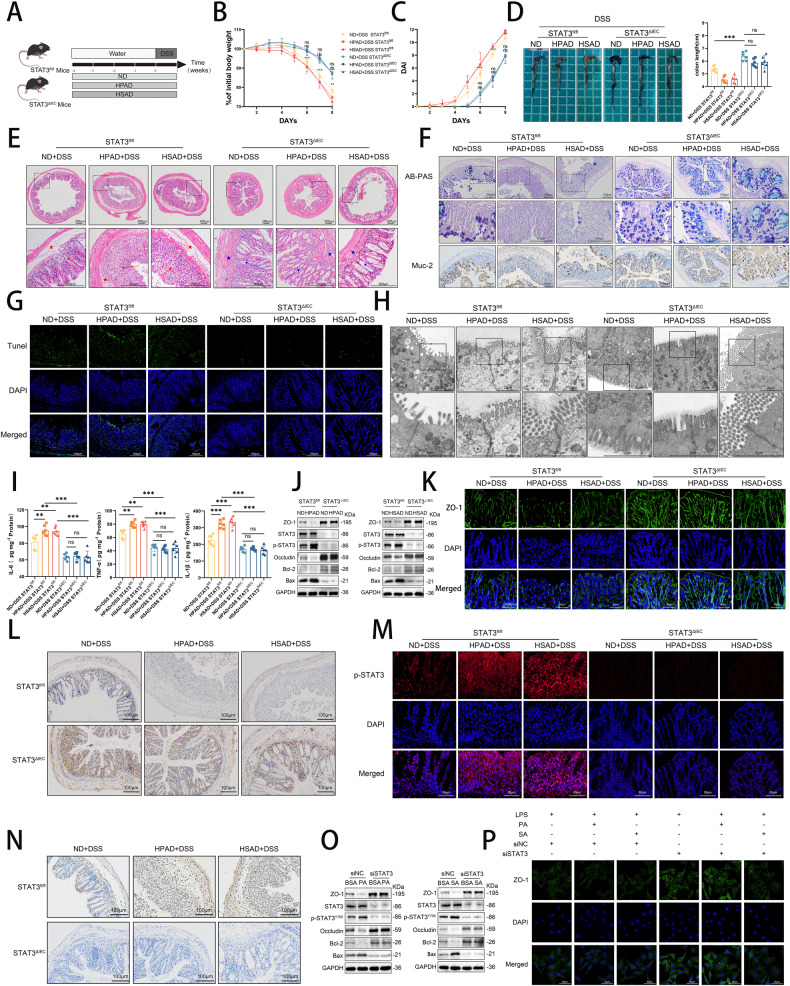
Suppression of STAT3 alleviates LCFAs-induced exacerbated inflammation and intestinal barrier damage in experimental colitis. **A** STAT3^fl/fl^ and STAT3^ΔIEC^ mice (*n* = 6) received ND, HPAD, and HSAD for 4 weeks, followed by continuous administration of 3% DSS in drinking water for 7 days; **B** Changes in mouse body weight from the first day of DSS intervention; **C** Changes in DAI in mice; **D** Representative colon morphology and length quantification; **E** Representative images of colon H&E staining. Mucosal erosions, ulceration, and mucosal atrophy (red arrows), and swollen submucosa with inflammatory cell infiltration (red star). Improvement noted in the loss of crypts (blue arrows), and mitigation of inflammatory cell infiltration (blue star); **F** Colonic tissue AB-PAS staining and IHC staining of MUC-2; **G** TUNEL staining; **H** Scanning electron microscopy images of mouse colonic tissue sections, magnifying tight junctions; **I** Levels of IL-6, TNF-α, and IL-1β in colonic tissues (*n* = 6); **J** Western blotting of colonic tissues; **K** Representative images of colonic tissue IF staining for ZO-1; **L** Colonic tissue IHC staining for ZO-1; **M** Colonic tissue IF staining for p-STAT3; **N** Colonic tissue IHC staining for p-STAT3. NCM460 cells were transfected with siSTAT3 or siNC and treated with LPS-PA, LPS-SA, or LPS-BSA for 24 h; **O** Results of western blotting; **P** IF staining of cells for ZO-1. Data were expressed as the mean ± SD. *P* values were determined using one-way or two-way ANOVA test.; ***p < 0.001, **p < 0.01, *p < 0.05.

In the subsequent phase of our investigation, we focused on the role of STAT3 signaling in exacerbating experimental colitis due to LCFA exposure (Fig. 5A). Our findings revealed that STAT3^ΔIEC^ mice exhibited milder colitis symptoms, such as reduced body weight (Fig. 5B), elevated DAI (Fig. 5C), and shortened colon length (Fig. 5D), compared to STAT3^fl/fl^ mice. Interestingly, these manifestations in STAT3^ΔIEC^ mice subjected to different dietary treatments displayed no significant differences, indicating that the absence of STAT3 in intestinal epithelial cells mitigated the detrimental effects of LCFAs on colitis development.

In addition, STAT3 knockout ameliorated various histological changes caused by the high-LCFA diet in colitis. This was evident from improvements observed in hematoxylin and eosin (H&E) staining (Fig. 5E), pathological scoring (Fig. S1A), AB-PAS staining, MUC-2 IHC staining (Fig. 5F), TUNEL staining (Figs. 5G, S1B), and scanning electron microscopy (Fig. 5H). At the same time, STAT3 knockout also reduced the differences in the expression of IL-6, TNF-α, and IL-1β (Fig. 5I), as well as ZO-1, occludin, BCL-2, and BAX in the colons of mice with colitis induced by different diets (Fig. 5J–N).

In our in vitro experiment, we employed siRNA to knock down STAT3. Our results showed that under LPS-induced inflammation, PA and SA had no evident effect on siSTAT3-transfected cells (Fig. 5O, P).

In summary, our results indicated that STAT3 knockdown can mitigate the elevation of inflammatory factors and intestinal barrier damage induced by LCFAs in inflammatory models, thereby suggesting the involvement of the STAT3 signaling pathway in the action of LCFAs.

### Dietary LCFAs promote the palmitoylation cycle of STAT3 in colitis

**Fig. 6:**
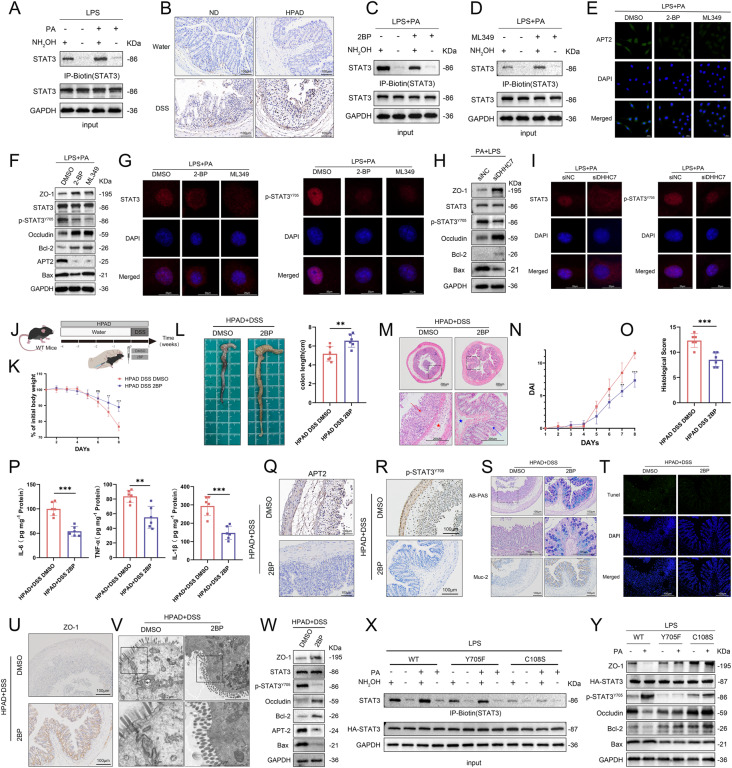
Dietary LCFAs promote the palmitoylation cycle of STAT3 in colitis. **A** The levels of palmitoylated STAT3 were assessed using the acyl–biotin exchange (ABE) method in LPS-BSA and LPS-PA-treated cells. **B** IHC staining of APT2 in colonic tissues of ND+water, HPAD+water, ND + DSS, and HPAD + DSS mice from the experiment shown in Fig. 2A. **C** Cells were pre-treated with 2-BP (50 μM) or DMSO for 24 h, followed by stimulation with LPS-PA for 24 h. The levels of palmitoylated STAT3 were measured using the ABE method. **D** Cells were pre-treated with ML349 (20 μM) or DMSO for 24 h, followed by stimulation with LPS-PA for 24 h. The levels of palmitoylated STAT3 were measured using the ABE method. NCM460 cells were pre-treated with 2-BP (50 μM) [24], ML349 (20 μM) [14] or DMSO (control) for 24 h, followed by stimulation with LPS-PA for 24 h. **E** IF staining to evaluate the effect of 2-BP and ML349 on APT2 in the LPS-PA cell model. **F** Western blotting to assess the impact of 2-BP, ML349, and the control group on the LPS-PA cell model. **G** IF staining was used to assess the influence of 2-BP and ML349 on nuclear translocation of STAT3 and p-STAT3. **H** NCM460 cells were transfected with siDHHC7 or siNC, followed by LPS-PA intervention for 24 h. Western blotting of cells. **I** IF staining was used to assess the impact of siDHHC7 on nuclear translocation of STAT3 and p-STAT3. **J** WT Mice (*n* = 6) were administered with HPAD for 4 weeks, followed by 7 days of 3% DSS in drinking water. Starting from the first day of DSS administration, the mice received daily intraperitoneal injections of 2-BP (50 mg kg^−1^) or vehicle control. **K** Changes in body weight. **L** Representative images and quantification of colon morphology and length in mice. **M** Representative images of colon H&E staining. Mucosal erosions, ulceration, and mucosal atrophy (red arrows), and swollen submucosa with inflammatory cell infiltration (red star). Improvement noted in the loss of crypts (blue arrows), and mitigation of inflammatory cell infiltration (blue star). **N** Changes in DAI. **O** Histological scoring of colonic tissue based on the morphology of colon sections. **P** Measurement of IL-6, TNF-α, and IL-1β levels in colonic tissue of mice (*n* = 6) using ELISA. **Q** IHC staining of APT2 in colonic tissue. **R** IHC staining of p-STAT3 in colonic tissue. **S** AB-PAS and IHC staining of MUC-2 in colonic tissue. **T** TUNEL staining of colonic tissue. **U** IHC staining of ZO-1 in colonic tissue. **V** Scanning electron microscopy images of colon tissue sections from mice, with magnified areas showing tight junctions. **W** Western blotting of colonic tissue. **X** NCM460 cells were transfected with HA-STAT3 (WT), HA-STAT3 (Y705F), and HA-STAT3 (C108S), and then stimulated with LPS-BSA or LPS-PA, and the levels of palmitoylated STAT3 were assessed using the ABE method. **Y** Western blotting of the cells. Data were expressed as the mean ± SD. *P* values were determined by 2-tailed Student *t* test.; ***p < 0.001, **p < 0.01, *p < 0.05.

The palmitoylation of STAT3 plays an important role in promoting its phosphorylation and nuclear translocation. In our study, we observed that PA treatment increased the levels of palmitoylated-STAT3 in the LPS-induced inflammation cell model (Fig. 6A). APT2 has been identified as a depalmitoylating enzyme for STAT3 [14], facilitating the nuclear translocation and transcriptional activity of phosphorylated STAT3. In vivo, we found higher APT2 expression in the inflammatory group compared to the physiological group, as well as in the HPAD-DSS group compared to the ND-DSS group (Fig. 6B). Indeed, these findings were also validated in our in vitro experiments (Fig. S2).

To explore the role of palmitoylation in STAT3 signaling, we treated cells with the palmitoylation inhibitor 2-Bromopalmitate (2-BP) [23–25] and the APT2 inhibitor ML349 [26, 27]. 2-BP downregulated the level of STAT3 palmitoylation induced by LPS-PA, whereas ML349 upregulated it (Fig. 6C, D). Both inhibitors, 2-BP and ML349, reduced the effects of PA on APT2, p-STAT3, ZO-1, occludin, BCL-2, and BAX expression (Fig. 6E–G, S3A). DHHC7 is the primary palmitoyltransferase of STAT3 [14]. Regarding the knockdown of DHHC7, we observed results that were consistent with the use of 2-BP and ML349 (Fig. 6H, I, S3B).

In in vivo experiments (Fig. 6J), 2-BP improved weight loss, reduced colon length shortening, and alleviated inflammation in colon tissue of mice (Fig. 6K–M). Moreover, 2-BP ameliorated the DAI and histopathological scores, while reducing IL-6, TNF-α, and IL-1β levels in the mouse colon (Fig. 6N–P). The colonic tissue of mice treated with 2-BP showed lower levels of APT2 and p-STAT3 compared to the control group (Fig. 6Q, R, S4A). Additionally, 2-BP alleviated the exacerbated intestinal mucosal barrier damage induced by HPAD (Fig. 6S–W, S4B, S4C).

In addition, our results revealed that the mutation of the STAT3 C108 site resulted in a considerable decrease in the level of palmitoylated-STAT3 in the inflammatory state. On the other hand, the mutation of the Y705 site led to a decrease, but it was relatively less significant compared to the C108 mutation. (Fig. 6X). Both mutations at the C108 and Y705 sites reduced the levels of p-STAT3 in the LPS-PA model, while also improving the integrity of the intestinal barrier and reducing the apoptotic indices (Fig. 6Y).

These results collectively indicated that LCFAs are involved in the palmitoylation cycle of STAT3, thereby promoting experimental colitis.

### Dietary LCFAs exacerbate colitis by regulating STAT3 palmitoylation through CD36-mediated endocytosis

**Fig. 7:**
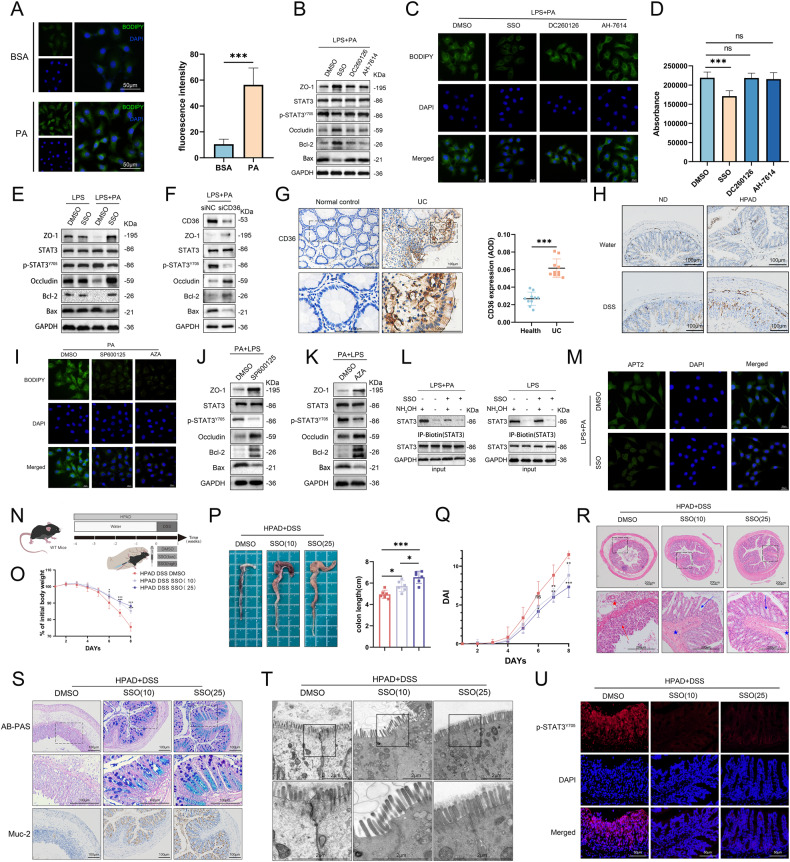
LCFAs are internalized into cells through CD36-mediated uptake and participate in the palmitoylation cycle of STAT3. **A** NCM460 cells were stimulated with PA or BSA for 24 h. BODIPY™ FL C12 was used for cell staining. Semi-quantitative analysis was performed on multiple fields (*n* = 9). **B** Pre-treatment with SSO (50 μM) [42], DC260126 (10 μM) [43], and AH-7614 (10 μM) [43, 44] was conducted for 1 h, with DMSO as the control. Subsequently, cells were co-treated with LPS and PA for 24 h. Western blotting of the cells. **C** BODIPY staining of cells. **D** FA uptake experiments were conducted to explore the impact of SSO, DC260126, and AH-7614 on FA uptake in NCM460 cells. **E** Cells were pre-treated with SSO or DMSO and subsequently stimulated with LPS alone or in combination with PA. Western Blotting. **F** NCM460 cells were transfected with siCD36 or siNC, followed by LPS-PA intervention for 24 h. Western Blotting. **G** IHC staining of CD36 in colonic tissues of UC patients and healthy controls. And AOD quantitative analysis on the IHC staining images of CD36 in UC (*n* = 10) and health (*n* = 10) groups. **H** IHC staining of CD36 in colonic tissues of ND+water, HPAD+water, ND + DSS, and HPAD + DSS mice of Fig. 2A. **I** NCM460 cells were pre-treated with SP600125 (20 μM) [15] or AZA (10 μM) [15] for 1 h, followed by PA intervention for 24 h. And BODIPY staining of each group. **J** NCM460 cells were pre-treated with SP600125 (20 μM) for 1 h, followed by co-treatment with LPS and PA for 24 h. Western Blotting. **K** NCM460 cells were pre-treated with AZA (10 μM) for 1 h, followed by co-treatment with LPS and PA for 24 h. Western Blotting. **L** The levels of palmitoylated STAT3 were assessed using the ABE method in LPS-PA-DMSO, LPS-BSA-SSO, LPS-BSA-DMSO, and LPS-BSA-SSO treated cells. **M** IF staining was used to assess the effect of SSO pre-treatment on the APT2 IF signal in cells stimulated with LPS-PA. **N** WT Mice (*n* = 6) were administered with HPAD for 4 weeks, followed by 7 days of 3% DSS in drinking water. Starting from the first day of DSS administration, the mice received daily intraperitoneal injections of SSO (10 mg kg^−1^), SSO (25 mg kg^−1^), or vehicle control. **O** Changes in body weight. **P** Representative images and quantification of colon morphology and length in mice. **Q** Changes in DAI in different groups of mice. **R** Representative images of colon H&E staining. Mucosal erosions, ulceration, and mucosal atrophy (red arrows), and swollen submucosa with inflammatory cell infiltration (red star). Improvement noted in the loss of crypts (blue arrows), and mitigation of inflammatory cell infiltration (blue star). **S** AB-PAS and IHC staining of MUC-2 in colonic tissue. **T** Scanning electron microscopy images of colon tissue sections from mice, with magnified areas showing tight junctions. **U** IF staining of p-STAT3 in colonic tissue. Data were expressed as the mean ± SD. *P* values were determined by one-way or two-way ANOVA test.; ***p < 0.001, **p < 0.01, *p < 0.05.

Next, we explored how dietary LCFAs are involved in the regulation of the intracellular STAT3 pathway. CD36 acted as an LCFA transporter [15, 16], while the G-protein coupled receptors, GRP120 and GRP40, bound to LCFAs, triggered diverse biological responses [28–32]. BODIPY staining revealed that PA upregulated the fluorescence intensity of cytoplasmic BODIPY (Fig. 7A). Subsequently, we pre-treated the LPS-PA model with the CD36 inhibitor (sulfosuccinimidyl oleate sodium, SSO), GPR40 inhibitor (DC260126), and GPR120 inhibitor (AH-7614). Western blotting showed that only SSO hindered the increase of p-STAT and BAX induced by PA, along with the decrease in ZO-1, occludin, and BCL-2 (Fig. 7B, S5A). Subsequently, we observed a considerable decrease in BODIPY fluorescence intensity (Fig. 7C) and FA uptake in cells (Fig. 7D) in the SSO group compared to the DMSO group, while no notable changes were observed in the DC260126 and AH-7614 groups. These findings suggested that PA may primarily exacerbate LPS-induced inflammation through CD36 rather than GPR40 and GPR120. The impact of SSO on the abovementioned proteins in the presence of LPS alone showed no significant difference compared to the control group, indicating that SSO may primarily exert its effects by inhibiting PA (Fig. 7E). Cells with siRNA-mediated CD36 knockdown exhibited similar results to SSO intervention (Fig. 7F).

Simultaneously, we found that CD36 expression was upregulated in UC patients compared to healthy controls (Fig. 7G). Similarly, CD36 expression was elevated in the HPAD-DSS group compared to the ND-DSS group (Fig. 7H). These findings suggested that LCFAs may also directly correlate to CD36 expression levels.

Moreover, we explored the involvement of VAVS and JNK in CD36-mediated endocytosis of FAs in adipocytes, as previously documented [15]. Inhibition of both VAVs (AZA) and JNK (SP600125) led to a decrease in cytoplasmic BODIPY fluorescence intensity (Fig. 7I). Additionally, these inhibitors reduced the elevation of p-STAT3 induced by PA in the inflammation model (Fig. 7J, K, S5B).

Then, we explored whether CD36 blockade affects STAT3 palmitoylation, and the results showed that both SSO and siCD36 reduced PA-induced palmitoylated STAT3 levels. SSO reduced APT2 levels in the LPS-PA model (Fig. 7L, M, S5C).

Next, we explored the in vivo effects of CD36 inhibition using SSO (Fig. 7N). Our findings showed that mice treated with both doses of SSO (10 mg kg^−1^ and 25 mg kg^−1^) experienced reduced weight loss (Fig. 7O) and colon shortening (Fig. 7P). SSO intervention alleviated the DAI in mice (Fig. 7Q), as well as the levels of inflammatory factors in colon tissue (Figure S6) and histopathological manifestations of colitis, including H&E staining alterations (Fig. 5R) and measurements of intestinal barrier function (Fig. 7S, T, S7). Additionally, histological staining indicated that SSO reduced the levels of p-STAT3 in the colon tissue of mice induced by HPAD + DSS (Fig. 7U, S8). Notably, a higher concentration of SSO exhibited even more remarkable anti-inflammatory effects compared to a lower concentration.

Collectively, our results provide evidence that dietary LCFAs exacerbate colitis by regulating STAT3 palmitoylation through CD36-mediated endocytosis.

## Discussion

The global incidence and prevalence of IBD have shown a concerning and continuous increase [33]. It is imperative to gain mechanistic insights into IBD to develop effective prevention and treatment strategies. Previous investigations have identified a Western diet as a risk factor for IBD [34]. This diet is characterized by its high content of saturated LCFAs, refined sugars, salt, and artificial sweeteners [2]. Earlier research has demonstrated that an HFD can promote experimental colitis [3, 4, 35]. However, the precise underlying mechanisms are still under investigation. In this study, we demonstrated an increased presence of LCFAs, particularly PA and SA, in the serum of UC patients. We further validated and elucidated their detrimental effects in promoting colonic inflammation in mice. Our findings indicated that LCFAs can enhance colonic inflammation by upregulating inflammatory factors, disrupting intestinal barrier integrity, and promoting intestinal epithelial cell apoptosis, thereby exacerbating DSS-induced experimental colitis.

The involvement and mechanisms of STAT3 in the occurrence and progression of IBD have been increasingly elucidated [36, 37]. The activation of the IL-6/STAT3 pathway has consistently been identified as a substantial component in colitis [12, 13]. Previous studies have revealed that PA can trigger STAT3 phosphorylation and exert biological effects in gastric cancer and prostate cancer models [21, 22]. Consistent with these findings, our results demonstrated that LCFAs upregulate the expression of p-STAT3 in the colitis model and promote the activation and nuclear translocation of STAT3. In STAT3^ΔIEC^ mice, the impact of LCFAs was found to be impeded by STAT3 deletion. Thus, our findings substantiate the hypothesis that LCFAs exacerbate colitis through the activation of the STAT3 pathway.

Protein S-acylation, specifically palmitoylation, is the fully reversible post-translational modification that regulates the characteristics and functions of various proteins. PA plays a consequential role in protein palmitoylation by conversion to palmitoyl-CoA, and serves as a substrate for protein S-acylation catalyzed by the DHHC family [38, 39]. Cys108 of STAT3 is palmitoylated by DHHC7, anchoring it to the cell membrane, and selective depalmitoylation of p-STAT3 by APT2 promotes the nuclear translocation of p-STAT3. This process is known as the palmitoylation cycle of STAT3 and has been shown to enhance Th17 differentiation and exacerbate colitis [14]. In our study, we demonstrated that PA upregulated the levels of palmitoylated STAT3 in LPS-induced inflammation models. APT2, an acyl protein thioesterase mainly present in cells, removes PA from cysteine residues of proteins and plays a substantial role in maintaining the palmitoylation cycle of STAT3 [40, 41]. We found that PA upregulated the levels of APT2 in inflamed colonic tissues and cells, providing evidence for the direct or indirect involvement of dietary-derived PA in the palmitoylation cycle of STAT3.

Our results showed that the broad-spectrum palmitoylation inhibitor 2-BP downregulated palmitoylated STAT3 in colitis cell models, while the selective APT2 inhibitor ML349 upregulated palmitoylated STAT3. Interestingly, both 2-BP and ML349, as well as knockdown of DHHC7, blocked the inflammatory upregulation induced by PA in in vitro experiments. This suggests that disrupting the normal operation of the palmitoylated STAT3 cycle can inhibit the pro-inflammatory effects of PA. These results also indicated that the palmitoylation–depalmitoylation cycle, rather than just palmitoylation alone, plays an important role in PA-mediated exacerbation of colitis. We obtained similar findings in in vivo experiments using 2-BP intervention.

The DHHC7-mediated palmitoylation of STAT3 at the Cys108 site promoted the membrane recruitment of STAT3 and phosphorylation at the Y705 site, while APT2 selectively depalmitoylated p-STAT3 and facilitated its nuclear translocation [14]. Our conclusion aligns with previous studies, suggesting that PA-LPS-induced palmitoylation of STAT3 can influence the phosphorylation level of STAT3. Importantly, phosphorylation of STAT3 is not a necessary condition for its palmitoylation, indicating that palmitoylation of STAT3 serves as an important upstream mechanism for STAT3 phosphorylation.

Previous studies have shown that LCFAs can enter cells through FA transporters, such as CD36 [15, 16], or exert various biological responses by binding to free LCFA receptors, such as the G protein-coupled receptors GRP120 and GRP40, on the cell membrane surface [28–32]. Our results revealed an upregulation of CD36 levels in colonic tissues of UC patients, and a high-LCFA diet increased CD36 expression in the colonic tissues of mice with colitis. In our in vivo experiments, we found that PA may exert its effects by being transported into cells via CD36, rather than by binding to GRP120 and GRP40 directly. The anti-inflammatory action of SSO is based on inhibition of PA uptake. In our in vivo experiments, both concentrations of SSO (10 mg kg^−1^ and 25 mg kg^−1^) effectively inhibited the promotional effect of HPAD on colitis. Additionally, pretreatment with inhibitors of the downstream molecules VAVS and JNK, which mediate CD36 endocytosis, successfully mitigated the promotional effect of PA on the LPS model. Furthermore, the inhibition of CD36 also successfully downregulated the palmitoylation of STAT3 induced by PA.

A limitation of our study is that we only explored the in-depth mechanisms of PA. PA is the primary lipid involved in the acylation of endogenous proteins. However, SA can also participate as an LCFA moiety in protein S-acylation. Further investigation is needed to determine whether SA enters cells and contributes to protein S-acylation. On the other hand, we observed that PA intervention upregulates the expression levels of APT2 in both in vivo and in vitro experiments. However, the specific regulatory mechanisms by which PA affects APT2 expression remain unclear and warrant further investigation.

In summary, dietary LCFAs, especially PA, can enter cells through CD36 uptake and participate in the palmitoylation cycle of STAT3, leading to the disruption of the intestinal barrier and exacerbation of colitis. These findings provide potential strategies for the prevention and treatment of IBD.

## Materials and methods

### Clinical information and human specimens

This study enrolled adult patients who were discharged from Renmin Hospital of Wuhan University with a confirmed diagnosis of UC between January 2021 and June 2023. The sample size was calculated a priori using GPower 3.1 software. The inclusion criteria were: 1) Definite UC diagnosis at discharge; 2) Serum testing for FFAs conducted during hospitalization; 3) Age over 14 years. The exclusion criteria were: 1) Incomplete clinical data; 2) Use of medications known to affect FFAs (e.g., β-receptor blockers, statins, insulin, and metformin); 3) Pregnancy or lactation; 4) History of metabolic syndrome, diabetes, or liver disease. Initially, 216 patients were identified, of whom 38 were excluded. Finally, 178 patients (118 males and 60 females) were included. Age and FFAs levels were collected for each patient. Disease activity status was assessed based on factors such as the erythrocyte sedimentation rate, stool frequency, rectal bleeding, hemoglobin levels, and presence of fever. Additionally, 178 individuals undergoing health examinations during the same period were selected as the control group. The severity of UC was classified based on the modified Truelove and Witts disease severity classification, including remission, mild activity, moderate activity, and severe activity. Furthermore, 10 serum samples were randomly collected from both the UC group and the healthy control group for GC-FID/MS Analysis of Serum FAs. Paraffin-embedded intestinal tissue (*n* = 10 for UC and n = 10 for normal tissues) was randomly collected from the department of pathology for IHC purposes. Ethical approval was granted by the Hospital Ethics Committee (WDRY2021-K025) and informed consent was obtained from all participants.

### Mice

C57BL/6 J WT mice were obtained from the Vital River Laboratory (Beijing, China) and acclimatized to the laboratory environment for one week. C57BL/6 J STAT3^ΔIEC^ (STAT3-deficient in intestinal epithelium cell) mice (Table S3, Figure S9) were generated by crossing STAT3^fl/fl^ mice and villin-cre mice, both of which were obtained from the Model Animal Research Center of Nanjing University (Nanjing, China). All mice were housed under specific pathogen-free conditions at the Animal Experiment Center of Renmin Hospital of Wuhan University (Wuhan, China). Male mice, aged 6–8 weeks and weighing between 18 and 22 g, were selected for the experiments. The animal experiments were carried out in accordance with the National Institutes of Health Guide for the Care and Use of Laboratory animals. Ethical approval for all animal experimentation protocols was obtained from the Laboratory Animal Welfare & Ethics Committee of Renmin Hospital of Wuhan University (PR China; approval number: 20211106). No statistical method was used to estimate the sample size for animal experiments.

### Dextran sulfate sodium (DSS)-induced colitis model

C57BL/6 J WT mice were randomly assigned to six groups (*n* = 6 per group) using a random number table, as follows: control mice fed with a normal diet (ND+Water), DSS-induced colitis mice fed with ND (ND + DSS), control mice fed with a high PA diet (HPAD+Water), DSS-induced colitis mice fed with HPAD (HPAD + DSS), control mice fed with a high SA diet (HSAD+Water), and DSS-induced colitis mice fed with HSAD (HSAD + DSS). STAT3^fl/fl^ mice and STAT3^ΔIEC^ mice were randomly divided into three groups (*n* = 6 per group), including DSS-induced colitis mice fed with ND, DSS-induced colitis mice fed with HPAD, and DSS-induced colitis mice fed with HSAD. The experimental diets were formulated by the Bio-PIKE Company (Chengdu, China). Detailed formulations are provided in Tables S4–5. After four weeks on the assigned diets, the mice received either autoclaved drinking water or 3% DSS (#160110, MP Biomedicals, CA, USA) for 7 days. Daily observations were made regarding body weight, stool consistency, and the presence of occult blood. On the last day of DSS administration, all mice were sacrificed, and their colons were removed and measured for length. Distal colonic tissues were fixed with 4% paraformaldehyde for 24 h and subsequently embedded in paraffin. For histological analysis, paraffin-embedded segments of colon tissue were sectioned at 3 μm thickness and stained with H&E. H&E-stained tissue sections were independently evaluated by two pathologists in a blinded manner. The criteria for the DAI and histological scoring are provided in Tables S6–7. No blind method is involved for other sections of the animal experiments.

### Cell culture and cell transfection

In previous studies, the human normal colon mucosal epithelial cell line NCM460 has been utilized to construct in vitro models of colitis [12, 20]. NCM460 cells were obtained from BeNa Culture Collection (Hebei China), authenticated by STR profiling and free of mycoplasma contamination. NCM460 cells were cultured in Dulbecco’s Modified Eagle Medium (DMEM) (Hyclone, Cytiva, MA, USA) supplemented with 10% v/v fetal bovine serum (FBS) (Gibico, USA) and 1% v/v penicillin/streptomycin (Gibico), at 37 °C, with 5% CO_2_. Cells were stimulated for 24 h with 1 μg/mL lipopolysaccharide (LPS) (*E. coli*, Sigma, Missouri, USA) in combination with 200 μM PA (#P5585, Sigma) and 100 μM SA (#S4751, Sigma) or bovine serum albumin (BSA) (#B2064, Sigma). For transfection, cells were transfected with Lipofectamine™ 3000 (#L3000008 Thermo Fisher Scientific, USA) in six-well plates, following the manufacturer’s instructions. siRNA-STAT3 was obtained from GenePharma (Shanghai, China), and siRNA-DHHC7 and siRNA-CD36 were obtained from TSINGKE (Beijing, China). All siRNA sequences are listed in Table S8. Additionally, the knockdown efficiency of siRNA was validated using quantitative polymerase chain reaction (qPCR) (Table S9, Fig. S10). All plasmids were designed and acquired from GenePharma.

### Targeted gas chromatography–flame ionization detector/mass spectrometry (GC-FID/MS) analysis of serum fatty acids

In this study, serum samples (50 μL) were subjected to a series of analytical procedures using a rigorous protocol. First, 10 μL of the internal standard (C17:0-D33 in acetonitrile, 0.2 mg mL^−1^) and 10 μL of 0.5% formic acid (Thermo Fisher Scientific) in water were added to the samples. The mixture was extracted with 300 μL of ethyl acetate (SCRC, Shanghai, China) and centrifuged at 3000 rpm for 10 min. The upper layer was collected, while the bottom layer underwent another extraction with 300 μL of ethyl acetate. The resulting extract was dried and re-dissolved in 50 μL of pyridine (J&K, Beijing, China). Derivatization was performed by adding 50 μL of N, O-bis(trimethylsilyl)-trifluoroacetamide (Sigma) and incubating the mixture at 60 °C for 30 min. The supernatant was then transferred into a sample vial for subsequent gas chromatography (GC) analysis. A set of 34 fatty acids (Table S10) was used as target analytes, procured from Sigma, J&K, and Cayman Chemical (MI, USA). The GC-FID/MS system utilized in this study consisted of an Agilent 7890B gas chromatography instrument coupled to an Agilent 5977B mass spectrometer (Agilent Technologies, USA). For the chromatographic separation, an Agilent HP-5 capillary GC column (30 m length, 0.25 mm internal diameter, 0.25 μm film thickness) was employed. The injection volume of the samples was set at 1 μL, and a splitter (1:10) was used to optimize the signal-to-noise ratio. The carrier gas used was helium. The temperature of the injection port was maintained at 280 °C. The GC temperature program started at 80 °C, ramped to 180 °C at a rate of 35 °C/min, further increased to 240 °C (5 °C/min), was held at 240 °C for 1.2 min, was raised to 260 °C (20 °C/min), held at 260 °C for 1 min, and finally elevated to 300 °C (30 °C/min), then held at 300 °C for 0.8 min. The mass spectra were acquired with an electron ionization voltage of 70 eV, and the molecular ions were selectively collected in selected ion monitoring mode. Serum total FFAs refers to the sum of the detected concentrations of free fatty acids above the detection limit for each sample.

### Mouse colitis models intervened with SSO and 2-BP

For the purpose of conducting inhibition studies, SSO (#HY-112847A, MCE, Shanghai, China) was administered via intraperitoneal injection to WT mice (*n* = 6) at a dose of 10 mg kg^−1^ mouse and 25 mg kg^−1^ on a daily basis during DSS initiation. As a control, WT mice were injected with 5% DMSO in corn oil (v/v). Additionally, 2-BP (#261604 Sigma, St. Louis, Missouri, USA) was intraperitoneally injected into WT mice (*n* = 6) at a dose of 50 mg/kg mouse on a daily basis during DSS initiation. Control mice received injections of 5% DMSO in corn oil (v/v).

### Western blotting

Total protein was extracted from tissues and cells with RIPA lysis buffer (Servicebio, Wuhan, China) with 1% protease inhibitors and phosphatase inhibitors (Servicebio). Nuclear and cytosolic proteins were extracted by a Nuclear and Cytoplasmic Protein Extraction Kit (#P0027, Beyotime, Shanghai, China). Protein lysis buffers were centrifuged at 4 °C, 12,000 rpm, for 10 min, and quantified with a BCA reagent kit (#P0012-1, Beyotime) after ultrasound. After separation by SDS-PAGE, samples were transferred to a Nitrocellulose membrane (#PN66485, Pall, USA) that was blocked in 5% BSA with TBST and incubated with primary antibody in TBST for 8–12 h. The membrane was washed three times in TBST and incubated with horseradish-peroxidase-conjugated secondary antibody in TBST for 1 h at room temperature. Protein levels were detected using enhanced chemiluminescence reagent (Biosharp, Hefei, China) and imaged with the ChemiDocTMXRS+ system (BIO-RAD, CA USA). The following primary antibodies were used: STAT3 (#79D7, CST, Boston, USA), P-STAT3 (#D3A7, CST), ZO-1 (#21773-1-AP, Proteintech, Wuhan, China), occludin (#13409-1-AP, Proteintech), Bcl-2 (#68103-1-Ig, Proteintech), Bax (#60267-1-Ig, Proteintech), CD36 (#ab252922, Abcam, MA, USA), APT2 (#ab151578, Abcam), LaminB (#12987-1-AP, Proteintech), HA tag (#51064-2-AP, Proteintech), and GAPDH (60004-1-Ig, Proteintech).

### IHC and IF

IHC staining was performed using an IHC Kit (#36312ES50, YEASEN, Shanghai, China) according to the manufacturer’s instructions. For IF, nonspecific binding sites were blocked with 15% goat serum in phosphate-buffered saline (PBS) for 1 h at room temperature prior to incubation with primary antibodies and diluted in 2% goat serum overnight at 4 °C. Fluorescence-conjugated secondary antibodies were then incubated for 1 h at 37 °C. Subsequently, the slides were sealed with anti-fluorescence quenching sealing solution containing DAPI (#36308ES11, YEASEN) and observed under a fluorescence microscope (Olympus, Tokyo, Japan). The primary antibodies used were as follows: ZO-1 (#ab221547, Abcam), STAT3 (#79D7, CST), P-STAT3 (#D3A7, CST), MUC-2 (#ab272692, Abcam), LYPLA2 (#sc-515061, Santa Cruz, CA,USA), CD36 (#ab252922, Abcam), CD36 (#ab252923, Abcam), CoraLite594-conjugated Goat Anti-Rabbit IgG(H + L) (#SA00013-4, Proteintech), and CoraLite488-conjugated Goat Anti-Rabbit IgG(H + L) (#SA00013-2, Proteintech).

### Electron microscopy (EM)

Fresh 1 mm long segments of the distal mouse colon tissue were fixed in EM fixative (#G1102, Servicebio) at 4 °C for 24 h, washed three times with 0.1 M phosphate buffer (PB PH7.4), and then post-fixed with 1% osmic acid (#18456, Ted Pella Inc, USA) in 0.1 M PB for 2 h at room temperature, protected from light. Following ethanol dehydration, the specimens were embedded in 812 embedding agent (# 90529-77-4, SPI, Shanxi, China) and then sectioned extra thinly and processed for EM following the standard procedure. Finally, samples were visualized and photographed using an HT7800 transmission electron microscope (HITACHI, Tokyo, Japan).

### Evaluation of intestinal barrier integrity in WT mouse model

Prior to oral administration, mice were subjected to fasting in the morning for four hours before gavage. For each mouse, a fresh solution containing 80 mg mL^−1^ of 4 kDa FITC-dextran (#CSN36366, CSNpharm, Shanghai, China) in sterile PBS was immediately prepared, taking precautions to avoid exposure to light. The administration of FITC-dextran was carried out orally by gavage at a dose of 0.8 mg g^−1^ body weight. One hour post-gavage, mouse abdominal fur was removed using an electric razor, and images of the abdominal area were captured using the In Vivo Imaging System (IVIS Lumina II, Caliper Life Science, USA) with a laser set at 470 nm and a resolution of 2.0 mm for in vivo fluorescence assessment. Four hours post-gavage, plasma samples were collected, and fluorescence intensity was measured using a Victor Nivo microplate reader (PerkinElmer, USA) (*λ*_ex_ = 485/*λ*_em_ = 530 nm).

### TUNEL and AB-PAS

Paraffin-embedded tissue samples were sectioned into 3 um thin slices. The TUNEL of apoptotic cells in mouse colon tissue was detected using a One Step TUNEL Apoptosis Assay Kit (#C1088, Beyotime), following the manufacturer’s protocol. TUNEL-positive cells were visualized under fluorescent microscopy to evaluate apoptotic and necrotic morphological alterations. AB-PAS (#G1285, Solarbio, Beijing, China) staining was performed in accordance with the manufacturer’s instructions.

### ELISA

The mouse colonic tissues were homogenized in cold PBS with 1% protease inhibitors and centrifuged at 12,000 rpm for 20 min at 4 °C. The protein concentration was quantified by BCA protein assay (#P0010, Beyotime). The concentrations of IL-6, TNF-α, and IL-1β in the supernatants were determined using mouse IL-6 ELISA kits (#M6000B R&D Systems, Minneapolis, Minnesota, USA), mouse TNF-α ELISA kits (#88-7324-88, Thermo Fisher Scientific), and mouse IL-1β ELISA kits (#88-7013-88, Thermo Fisher Scientific), following the manufacturer’s instructions. IL-6 levels in cell culture supernatants were determined using a human IL-6 ELISA kit (#88-7066-88, Thermo Fisher Scientific), according to the manufacturer’s instructions.

### FA uptake

To investigate FA uptake in ncm460 cells, a pretreatment step was performed in serum-free medium for 1 h. Subsequently, the cells were exposed to SSO (50 μM), AH-7614 (10 μM) (#HY-19996, MCE), DC260126 (10 μM) (#HY-101906, MCE), or DMSO as a control for 1 h. The uptake of fatty acids was assessed using the Fatty Acid Uptake kit (#MAK156, Sigma), following the manufacturer’s instructions. The fluorescence intensity ((λ_ex_ = 485/ λ_em_ = 515 nm) was measured after a 30-min incubation using a Victor Nivo microplate reader (PerkinElmer, MA, USA).

### Determination of FFA contents

Mouse serum FFA was measured using a Free Fatty Acid Quantitation Kit (#MAK044, Sigma) with standardized protocols and determined via a colorimetric method using a Victor Nivo microplate reader (PerkinElmer)

### BODIPY

Cells were plated in a 12-well plate paved with sterile slips and were given PA (200 μM) and BSA (control) for 24 h. To study the impact of different inhibitors on the cellular FA uptake, cells were preincubated with SSO (50 μM), AH-7614 (10 μM), DC260126 (10 μM), and DMSO (control) for 1 h at 37 °C. Subsequently, the cells were stimulated for 24 h with LPS in combination with 200 μM PA. After washing with PBS three times, the cells were stained with 40 μM BODIPY™ FL C_12_ (#D3822, Thermo Fisher Scientific) for 15 min at 37 °C and fixed with 4% paraformaldehyde, and a nuclear stain (DAPI) was performed.

### Acyl–biotin exchange (ABE)

ABE was carried out according to the method described by a previous study [14]. Briefly, the cell samples were lysed with 1 mL lysis buffer A for 30 min on ice and sonicated for 1 min. Following centrifugation at 12,000 rpm at 4 °C for 20 min, the supernatants were collected for protein quantification using the BCA method. Proteins (10 μg) were precipitated three times with methanol/chloroform/water (v/v 1:4:3) and redissolved by adding 300 μL 4SB solution containing 10 mM N-ethylmaleimide (NEM) (#N808610, Macklin Biochemical Technology Shanghai, China) for 10 min at 37 °C. The samples were incubated with lysis buffer B overnight at 4 °C. The proteins were precipitated with methanol/chloroform/water three times again, then redissolved in 4SB solution. The solution was divided into two portions, which were supplemented further with +/-NH_2_OH buffer, and incubated for 1 h in RT. After protein precipitation, the samples were redissolved in 4SB and incubated with low HPDP-Biotin buffer (#B871617, Macklin Biochemical Technology) for 1 h in RT. Then, the proteins were dissolved with lysis buffer C for 30 min at RT and centrifuged at 13,000 rpm for 1 min. The supernatant was incubated for 1.5 h at 37 °C with avidin-conjugated magnetic beads (#88816, Thermo Fisher Scientific). After washing three times with buffer, the samples were mixed with SDS-PAGE reducing buffer and heated at 100 °C for 5 min for use for SDS-PAGE electrophoresis. Details of all formulations are shown in Table S11.

### Statistical analyses

Statistical analyses and graph generation were performed using GraphPad Prism 8, except for the ROC analysis, which was conducted using SPSS27. Data normality was assessed using the Shapiro-Wilk test, and homogeneity of variances was tested using Levene’s test. A Student’s *t* test or Mann–Whitney *U* test was employed to compare two variables, while analysis of variance or the Kruskal–Wallis test was used for comparing three or more variables. Spearman’s correlation analyses were performed for correlation analysis in this study. *P* < 0.05 was designated as significant.

### Reporting summary

Further information on research design is available in the Nature Research Reporting Summary linked to this article.

### Supplementary information

Supplemental Material

Western blots

Reporting Summary

## Data Availability

All data relevant to the study are included in the article or uploaded as supplementary information.
