# Supplementary material for: Dietary long-chain fatty acids promote colitis by regulating palmitoylation of STAT3 through CD36-mediated endocytosis
Source: Cell Death Dis. 2024 Jan 17;15(1):60. doi: 10.1038/s41419-024-06456-5 (PMC10794235; doi:10.1038/s41419-024-06456-5)
Supplement: Supplementary file 1 — Supplemental Material [file 41419_2024_6456_MOESM1_ESM.docx]

**Supplementary Materials**

**Table S1.** Comparison of baseline levels and FFA levels between UC patients and the healthy control group. The data of age and FFA were presented as median [range], and the P value was determined using unpaired two-tailed Mann-Whitney test.

|  | Health (n=178) | UC (n=178) | P value |
| --- | --- | --- | --- |
| Male | 65.7% | 66.3% | 0.911 |
| Female | 34.3% | 33.7% | 0.911 |
| Age | 45.50 [38.75-57.00] | 43 [33.75-56.00] | 0.086 |
| FFA (mM) | 0.16 [0.09-0.29] | 0.33 [0.15-0.66] | <0.001 |

**Table S2.** In targeted metabolomics, the levels of various FAs in the healthy control group and UC patients were quantified and compared. The data were presented as mean ± SD, and the P value was determined using t-tests.

| Free Fatty Acids | Health (n=10) (mean±SD) | UC (n=10) (mean±SD) | P value |
| --- | --- | --- | --- |
| C10:0 | 1.15±0.57 | 1.99 ±0.95 | 0.0412 |
| C12:0 | 1.09±0.61 | 9.16±11.26 | 0.0065 |
| C14:0 | 4.48±2.18 | 11.01±7.23 | 0.0102 |
| C14:1 | 0.33±0.23 | 1.51±1.5 | 0.0064 |
| C15:0 | 0.86±0.47 | 1.33±0.60 | 0.0584 |
| C16:0 | 89.63±30.10 | 183.17±58.06 | 0.0003 |
| C16:1 | 8.50±3.89 | 28.81±23.34 | 0.0082 |
| C17:0 | 2.00±1.31 | 2.40±0.87 | 0.1857 |
| C18:3n6c | 0.80±0.32 | 1.51±1.25 | 0.2861 |
| C18:2n6c | 85.42±31.97 | 186.30±73.60 | 0.0018 |
| C18:1n9c | 137.75±129.55 | 259.58±111.36 | 0.0102 |
| C18:0 | 60.39±19.36 | 96.96±18.96 | 0.0004 |
| C18:3n3c | 10.48±5.43 | 21.01±8.60 | 0.0052 |
| C18:1n9t | 11.90±10.36 | 19.32±8.11 | 0.0156 |
| C20:4n6 | 22.74±2.56 | 25.10±5.58 | 0.2461 |
| C20:3n6 | 2.22±0.21 | 2.46±0.65 | 0.7624 |
| C20:5n3 | 3.44±0.95 | 3.27±1.16 | 0.7313 |
| C20:1 | 4.83±4.98 | 5.85±2.66 | 0.0156 |
| C20:2n6 | 3.02±2.17 | 4.36±1.64 | 0.0343 |
| C20:0 | 2.25±3.12 | 1.39±0.71 | 0.8798 |
| C22:6n3 | 18.85±2.56 | 20.32±4.75 | 0.3999 |
| C22:5n3 | 1.60±0.58 | 2.10±0.81 | 0.1301 |
| C22:5n6 | 3.24±0.84 | 3.39±0.63 | 0.6745 |
| C22:4n6 | 2.56±0.48 | 3.12±0.73 | 0.0494 |
| C22:1 | 1.43±2.11 | 1.23±0.69 | 0.2265 |
| C22:0 | 1.03±0.87 | 0.70±1.09 | 0.0208 |
| C24:1 | 1.55±0.74 | 1.70±0.43 | 0.5802 |
| C24:0 | 1.46±0.80 | 0.93±1.23 | 0.0046 |


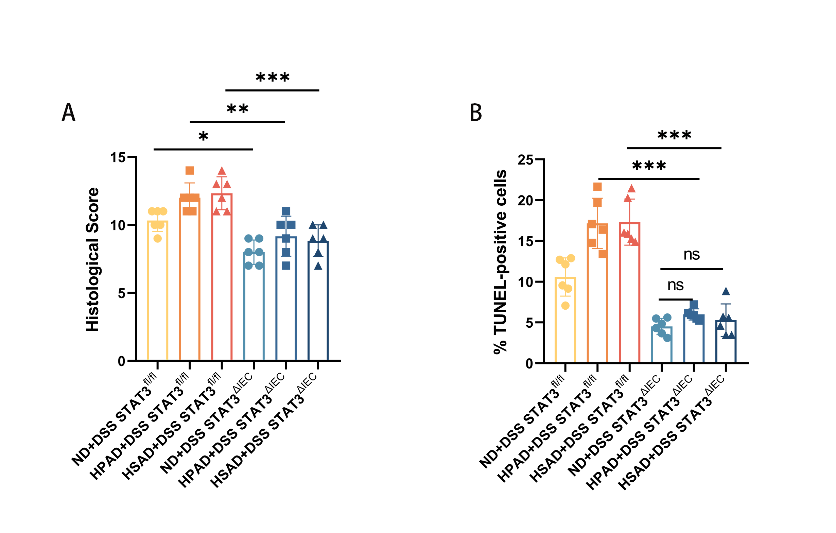


**Figure S1** Male SPF STAT3^fl/fl^ and STAT3^ΔIEC^ mice (n=6) received ND, HPAD, and HSAD for 4 weeks, followed by continuous administration of 3% DSS in drinking water for 7 days. (A) Histological scoring of colon sections. (B) statistical analysis of TUNEL-positive cells in TUNEL staining of colon tissue. Statistical significance was determined using one-way ANOVA test. ***p < 0.001, **p < 0.01, *p < 0.05.

**
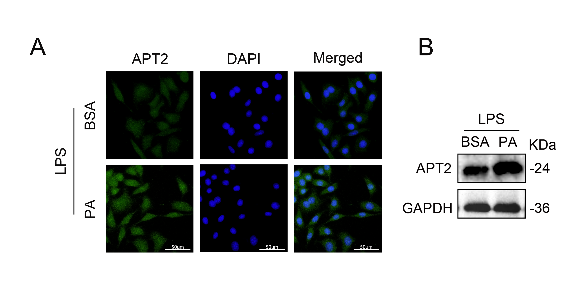
**

**Figure S2** (A) IF staining of APT2 was conducted in cells treated with LPS-BSA and LPS-PA. (B) WB analysis was performed to determine the expression levels of APT2 in cells treated with LPS-BSA and LPS-PA.

**
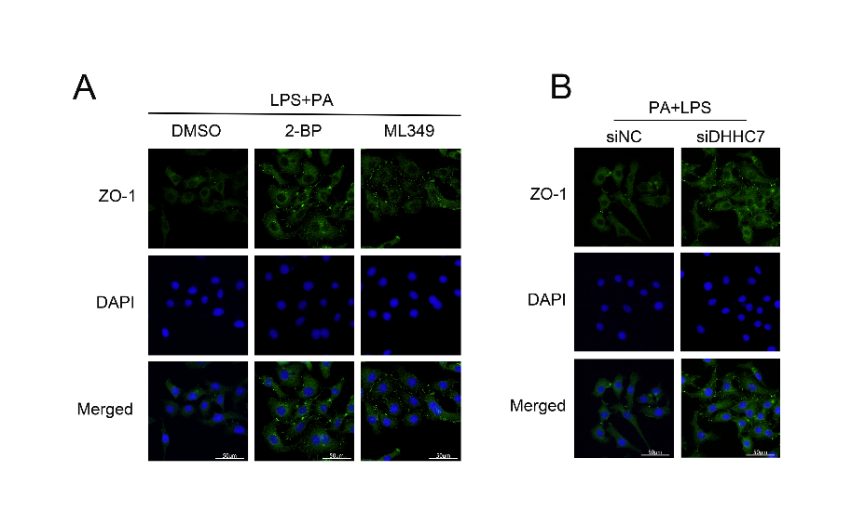
**

**Figure S3** (A) NCM460 cells were pre-treated with 2-BP, ML349 or DMSO (control) for 24 hours, followed by stimulation with LPS-PA for 24 hours. IF staining was performed to examine the changes in ZO-1 expression. (B) NCM460 cells were transfected with siDHHC7 or siNC, followed by LPS-PA intervention for 24 hours. IF staining was conducted to evaluate the changes in ZO-1 expression.

**
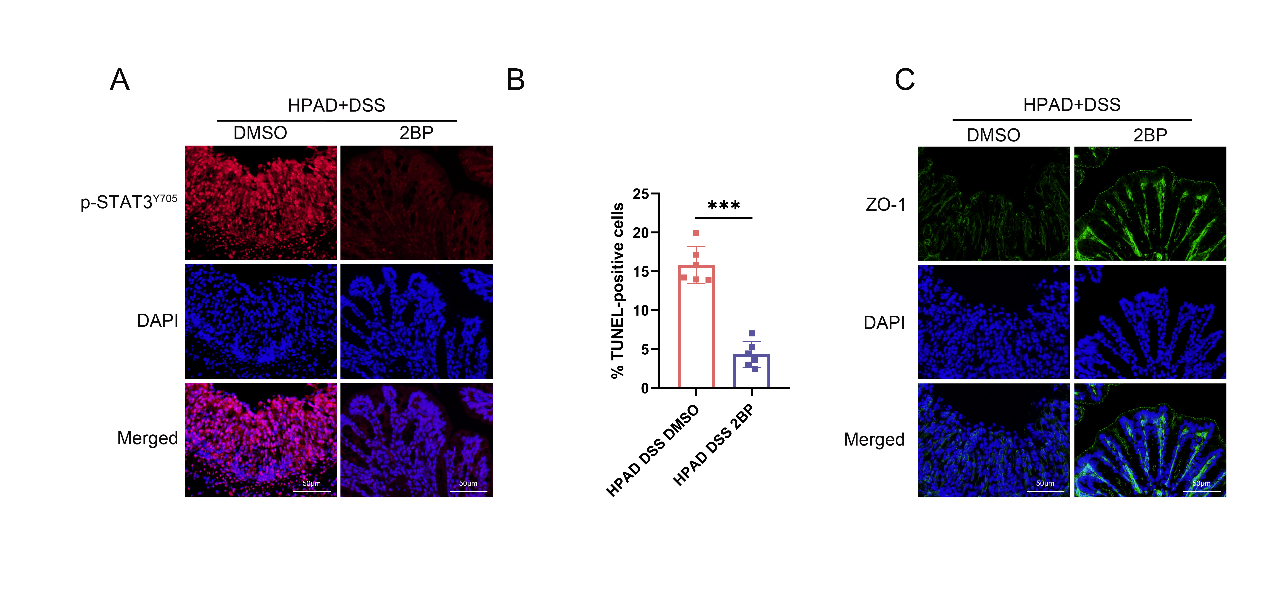
**

**Figure** **S4** SPF C57BL6J male WT mice (n=6) were administered with HPAD for 4 weeks, followed by 7 days of 3% DSS in drinking water. Starting from the first day of DSS administration, the mice received daily intraperitoneal injections of 2BP (50 mg/kg)^1^ or vehicle control (corn oil containing 5%DMSO). (A) IF staining of p-STAT3 in colonic tissue. Statistical significance was determined using 2-tailed Student t test. (C)Statistical analysis of TUNEL-positive cells in Figure6T. D) Representative images of IF staining of ZO-1 in colonic tissue. Statistical significance was determined using 2-tailed Student t test. ***p < 0.001, **p < 0.01, *p < 0.05.

**
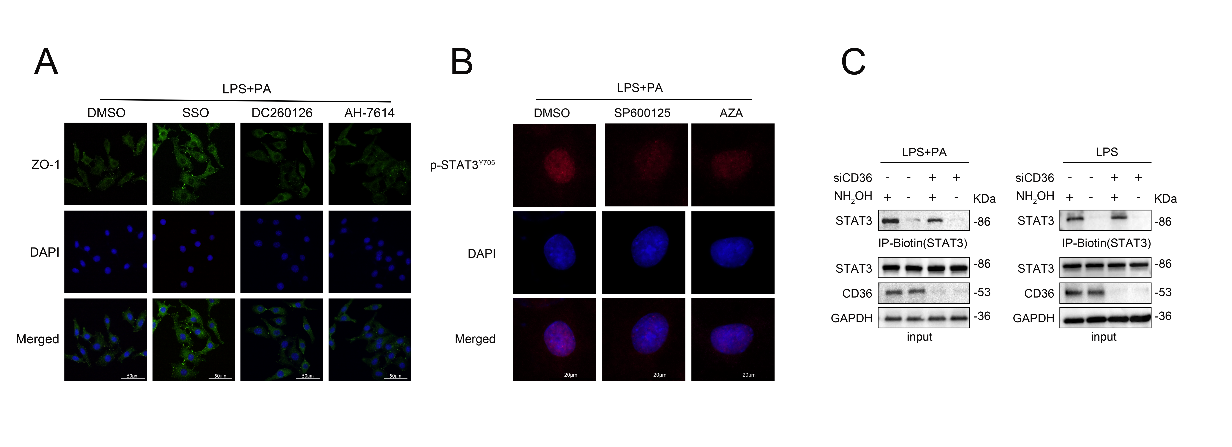
**

**Figure S5** (A) Pre-treatment with SSO, DC260126, and AH-7614 was conducted for 1 hour, with DMSO as the control. Subsequently, cells were co-treated with LPS and PA for 24 hours. IF staining images of ZO-1 in cells. (B) IF staining was performed to examine the impact of pre-treatment with SP600125 and AZA on p-STAT3 nuclear translocation in cells stimulated with LPS and PA. (C) The levels of palmitoylated STAT3 were assessed using the ABE method in LPS-PA-siNC, LPS-BSA-siCD36, LPS-BSA-siNC and LPS-BSA-siCD36-treated cells.

**
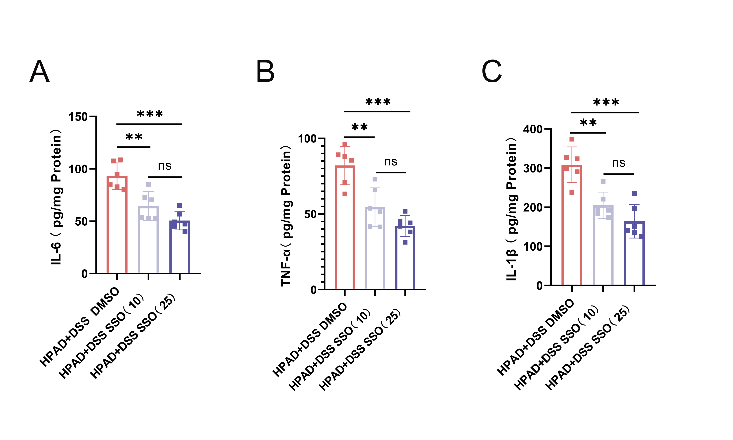
**

**Figure S6** WT Mice (n=6) were administered with HPAD for 4 weeks, followed by 7 days of 3% DSS in drinking water. Starting from the first day of DSS administration, the mice received daily intraperitoneal injections of SSO (10 mg/kg), SSO (25 mg/kg), or vehicle control (5%DMSO in corn oil). Measurement of IL-6, TNF-α, and IL-1β levels in colonic tissue of mice (n=6) using ELISA. Statistical significance was determined using 2-tailed Student t test. ***p < 0.001, **p < 0.01, *p < 0.05.

**
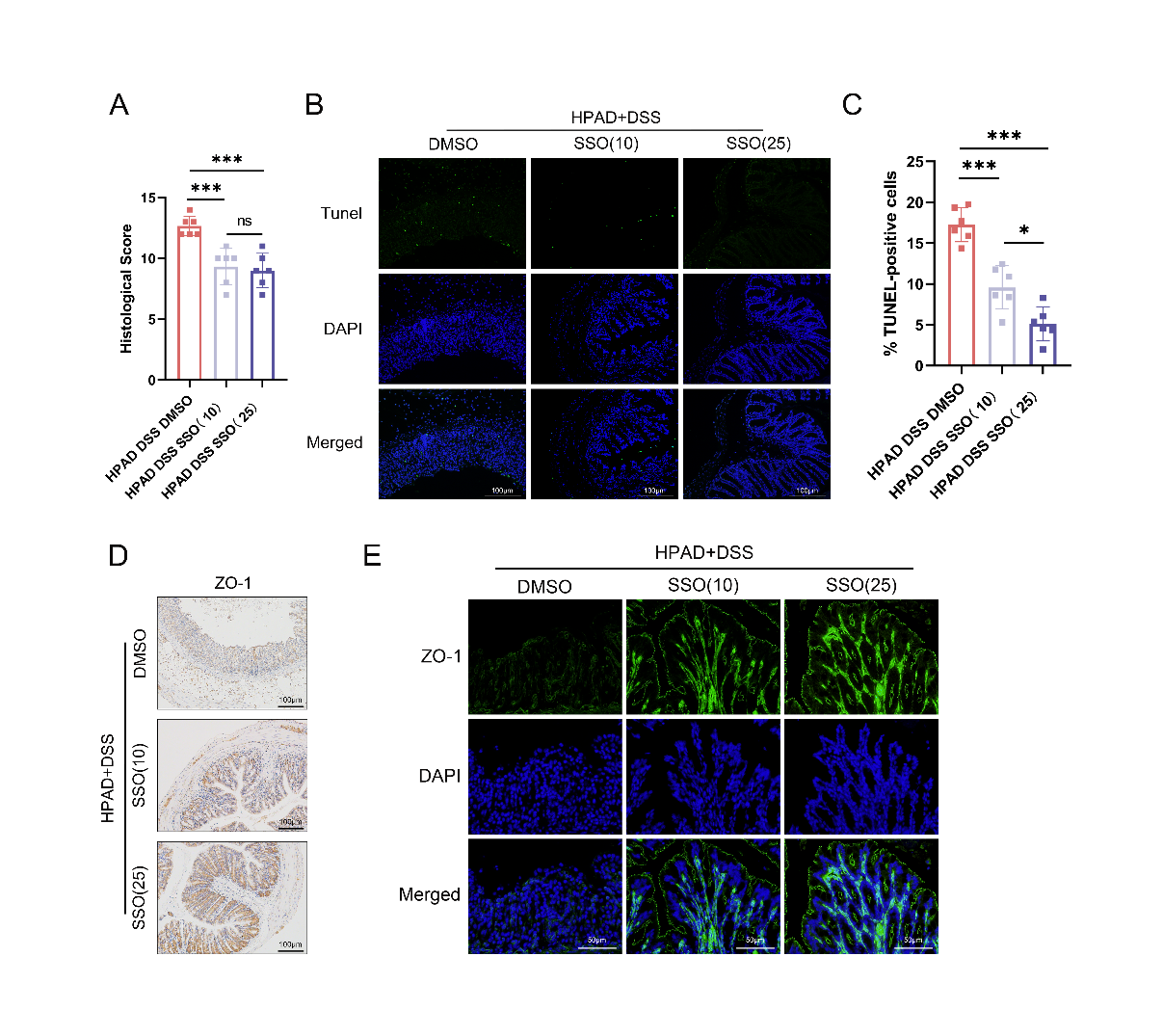
**

**Figure** **S7** Mice (n=6) were administered with HPAD for 4 weeks, followed by 7 days of 3% DSS in drinking water. Starting from the first day of DSS administration, the mice received daily intraperitoneal injections of SSO (10 mg/kg), SSO (25 mg/kg), or vehicle control (corn oil containing 5%DMSO). (A) Histological scoring of colonic tissue based on the morphology of colon sections. (C) TUNEL staining of colonic tissue and (C) statistical analysis of TUNEL-positive cells. (D) IHC staining of ZO-1 in colonic tissue. (E) Representative images of IF staining of ZO-1 in colonic tissue. Statistical significance was determined using one-way ANOVA test. ***p < 0.001, **p < 0.01, *p < 0.05.

**
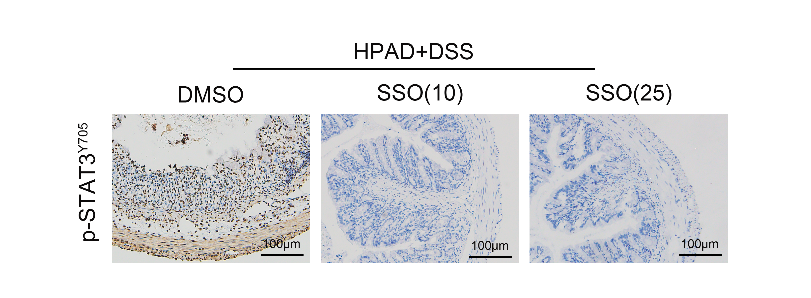
**

**Figure S8** WT Mice (n=6) were administered with HPAD for 4 weeks, followed by 7 days of 3% DSS in drinking water. Starting from the first day of DSS administration, the mice received daily intraperitoneal injections of SSO (10 mg/kg), SSO (25 mg/kg), or vehicle control (corn oil containing 5%DMSO). IHC staining of p-STAT3 in colonic tissue.

| Target | | Sequence 5’-3’ |
| --- | --- | --- |
| STAT3 flox | sense | TTGACCTGTGCTCCTACAAAAA |
|  | antisense | CCCTAGATTAGGCCAGCACA |
| Villin Cre | sense | ATTTGCCTGCATTACCGGTC |
|  | antisense | ATCAACGTTTTCTTTTCGG |

**Table S3.** primer for Mouse genotypes


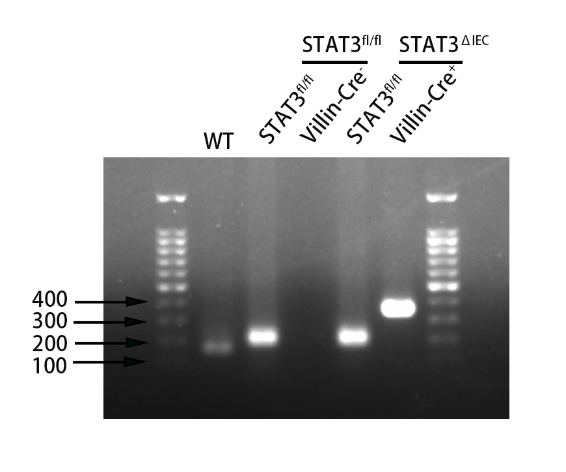


**Figure S9.** Representative PCR images from WT, STAT3^△IEC^ and STAT3^fl/fl^ mice.

**Table S4.** The composition of the diet for mice^2^

| Ingredients | ND | | HSAD | | HPAD | |
| --- | --- | --- | --- | --- | --- | --- |
|  | gm | kcal | gm | kcal | gm | kcal |
| Casein | 200 | 800 | 200 | 800 | 200 | 800 |
| L-cystine | 3 | 12 | 3 | 12 | 3 | 12 |
| Corn starch | 397.486 | 1589.944 | 287.486 | 1149.944 | 287.486 | 1149.944 |
| sucrose | 100 | 400 | 100 | 400 | 100 | 400 |
| Maltodextrin | 132 | 528 | 132 | 528 | 132 | 528 |
| Fiber | 50 | 0 | 50 | 0 | 50 | 0 |
| Soybean oil | 70 | 630 | 30 | 270 | 30 | 270 |
| Lard | 0 | 0 | 150 | 1350 | 0 | 0 |
| Palm oil | 0 | 0 | 0 | 0 | 150 | 1350 |
| Tert-butylhydroquinone | 0.014 | 0 | 0.014 | 0 | 0.014 | 0 |
| Mineral mixture (S10022G) | 35 | 0 | 35 | 0 | 35 | 0 |
| Vitamin mixture (V10037) | 10 | 40 | 10 | 40 | 10 | 40 |
| Choline bitartrate | 2.5 | 0 | 2.5 | 0 | 2.5 | 0 |
| Pigment (RED) | 0 | 0 | 0.05 | 0 | 0 | 0 |
| Pigment (BLUE) | 0 | 0 | 0 | 0 | 0.05 | 0 |
| Total amount | 1000 | 4000 | 1000.05 | 4550 | 1000.05 | 4550 |

**Table S5.** The ingredients of the diet for mice

| Ingredients | ND | | HSAD | | HPAD | |
| --- | --- | --- | --- | --- | --- | --- |
|  | gm% | Kcal% | gm% | Kcal% | gm% | Kcal% |
| Protein | 20 | 20.3 | 20 | 17.8 | 20 | 17.8 |
| Carbohydrates | 64 | 63.9 | 53 | 46.5 | 53 | 46.5 |
| Fat | 7 | 15.8 | 18 | 35.6 | 18 | 35.6 |

**Table S6.** Scoring system for Disease Activity Index (DAI)

| Score | Weight loss | Stool consistency | Blood stool |
| --- | --- | --- | --- |
|  |  |  |  |
| 0 | no loss | normal | no blood |
| 1 | 1-5% | loose stool | no blood |
| 2 | 5-10% | watery diarrhea | Presence of blood |
| 3 | 10-20% | slimy diarrhea, little blood | Presence of blood |
| 4 | ＞20% | severe watery diarrhea with blood | gross bleeding |

**Table S7.** The histopathological scoring in a blinded manner

| Score | Extent of Injury | Glandular Mucosal Atrophy | Tissue Damage | Inflammatory Cell Infiltration |
| --- | --- | --- | --- | --- |
| 0 | N/A | None | No mucosal injury | Rare inflammatory cells in the lamina propria |
| 1 | ≤25% | Mild | Discrete epithelial lesions | Increased number of inflammatory cells in the lamina propria |
| 2 | ≤50% | Moderate | Superficial mucosal erosion or focal ulceration | Confluent inflammatory cells extending into the submucosa |
| 3 | ≤75% | Moderate | Extensive mucosal damage extending into deeper layers of the intestinal wall | Transmural extension of infiltrates |
| 4 | ≤100% | Severe | - | - |

The overall histopathological score is determined by summing the scores from each category.

**Table S8**. siRNA sequences are listed:

| Target | | Sequence 5’-3’ |
| --- | --- | --- |
| h-siSTAT3 | sense | GGGACCUGGUGUGAAUUAUTT |
|  | antisense | AUAAUUCACACCAGGUCCCTT |
| h-siCD36-1 | sense | CGACAUGAUUAAUGGUACA(dT)(dT) |
|  | antisense | UGUACCAUUAAUCAUGUCG(dT)(dT) |
| h-siCD36-2 | sense  antisense | GGACCAUUGGUGAUGAGAA(dT)(dT) |
|  |  | UUCUCAUCACCAAUGGUCC(dT)(dT) |
| h-siCD36-3 | sense | CACUAUCAGUUGGAACAGA(dT)(dT) |
|  | antisense | UCUGUUCCAACUGAUAGUG(dT)(dT) |
| h-siDHHC7-1 | sense | CGGAGAUCGAGCGAUUGAA(dT)(dT) |
|  | antisense | UUCAAUCGCUCGAUCUCCG(dT)(dT) |
| h-siDHHC7-2 | sense | GAACAAUUGUGUAGGAGAA(dT)(dT) |
|  | antisense | UUCUCCUACACAAUUGUUC(dT)(dT) |
| h-siDHHC7-3 | sense | GGAGCCGAGUCUGUGCAAA(dT)(dT) |
|  | antisense | UUUGCACAGACUCGGUUCC(dT)(dT) |
| h-siNC | sense | UUCUUCGAACGUGUCACGUTT |
|  | antisense | ACGUGACACGUUCGGAGAATT |

**Table S9.** The primer sequences used for quantitative polymerase chain reaction (qPCR) are listed as follows.

| Target | | Sequence 5’-3’ |
| --- | --- | --- |
| h-STAT3 | sense | CCTCTGCCGGAGAAACAGT |
|  | antisense | CATTGGGAAGCTGTCACTGTAG |
| h-CD36 | sense | TGGTGCTGTCCTGGCTGTGT |
|  | antisense | TTGCTGCTGTTCATCATCACTTCCT |
| h-DHHC7 | sense | CGTCATGCTGCTGCCTTCCAA |
|  | antisense | CCCTCACCACAAATGCCCACAA |
| h-GAPDH | sense | GACAAGCTTCCCGTTCTCAG |
|  | antisense | GAGTCAACGGATTTGGTCGT |


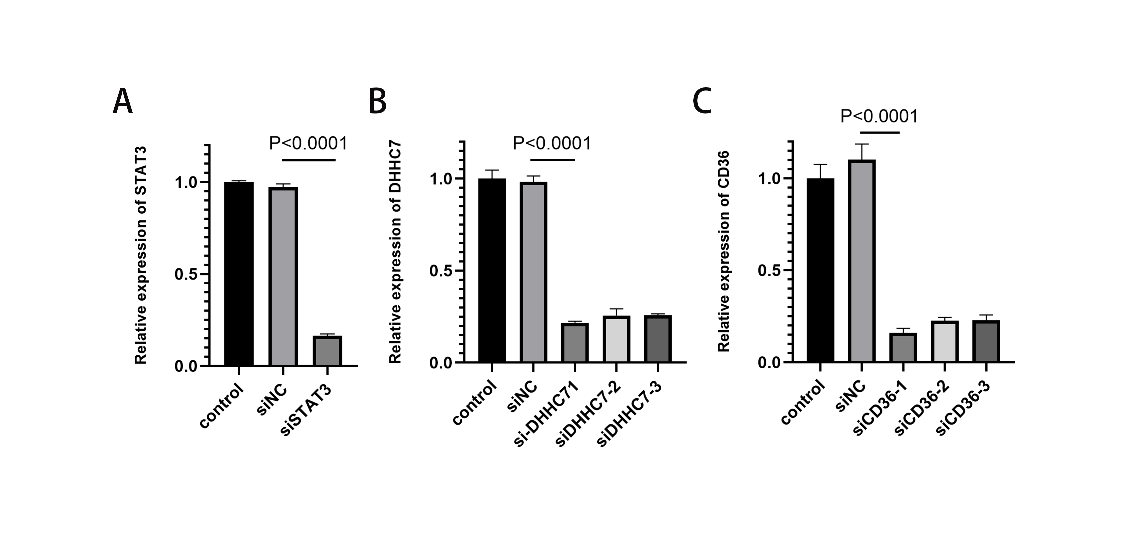


**Figure S10.** (A)NCM460 cells transfected with nontargeting siRNAs (siNC) and STAT3-targeting siRNA (siSTAT3), the mRNA expression of STAT3 was detected. (B) NCM460 cells transfected with siNC, DHHC7-targeting siRNA (si DHHC7-1), siDHHC7-2 and siDHHC7-3, the mRNA expression of DHHC7 was detected. (C) NCM460 cells transfected with siNC, siCD36-1, siCD36-2 and siCD36-3, the mRNA expression of CD36 was detected.

**Table S10.** 34 fatty acid analytes

|  | **Abbreviation** | **Common Name** | **Fatty acid** |
| --- | --- | --- | --- |
| 1 | C10:0 | Capric acid | Decanoic acid |
| 2 | C11:0 | Undecanoic acid | Undecanoic acid |
| 3 | C12:0 | Lauric acid | Dodecanoic acid |
| 4 | C13:0 | Tridecanoic acid | Tridecanoic acid |
| 5 | C14:1 | Myristoleic acid | *Cis*-9-tetradecenoic acid |
| 6 | C14:0 | Myristic acid | Myristic acid |
| 7 | C15:0 | Pentadecanoic acid | Pentadecanoic acid |
| 8 | C16:1 | Palmitoleic acid | *Cis*-9-Hexadecenoic acid |
| 9 | C16:0 | Palmitic acid | Hexadecanoic acid |
| 10 | C17:0 | Heptadecanoic acid | Heptadecanoic acid |
| 11 | C18:3n6c | γ-Linolenic acid | *Cis*-6,9,12-Octadecatrienoic acid |
| 12 | C18:2n6c | Linoleic acid | *Cis*-9,12-Octadecenoic acid |
| 13 | C18:1n9c | Oleic acid | *Cis*-9-Octadecenoic acid |
| 14 | C18:1n9t | Elaidic acid | *Trans*-9-Octadecenoic acid |
| 15 | C18:0 | Stearic acid | Octadecanoic acid |
| 16 | C18:3n3c | Linolenic acid (α-Linolenic acid) | *Cis*-9,12,15-Octadecatrienoic acid |
| 17 | C20:4n6 | Arachidonic acid (ARA) | *Cis*-5,8,11,14-Eicosapentaenoic acid |
| 18 | C20:5n3 | Eicosapentaenoic acid (EPA) | *Cis*-5,8,11,14,17-Eicosapentaenoic acid |
| 19 | C20:3n6 | γ-Homolinolenic acid | *Cis*-8,11,14-Eicosatrienoic acid |
| 20 | C20:1 | - | *Cis*-11-Eicosenoic acid |
| 21 | C20:3n3 | - | *Cis*-11,14,17-Eicosatrienoic acid |
| 22 | C20:2n6 | Eicosadienoic acid | *Cis*-11,14-Eicosadienoic acid |
| 23 | C20:0 | Arachidic acid | Icosanoic acid |
| 24 | C22:6n3 | Docosahexaenoic acid (DHA) | *Cis*-4,7,10,13,16,19-Docosahexaenoic acid |
| 25 | C21:0 | - | Heneicosanoic acid |
| 26 | C22:5n3 | Docosapentaenoic acid (DPA) | *Cis*-7,10,13,16,19-Docosapentaenoic acid |
| 27 | C22:5n6 | - | *Cis*-4,7,10,13,16-Docosapentaenoic acid |
| 28 | C22:4n6 | - | *Cis*-7,10,13,16-Docosatetraenoic acid |
| 29 | C22:2n6 | - | *Cis*-13,16-Docosadienoic acid |
| 30 | C22:1 | Erucic acid | *Cis*-13-Docosenoic acid |
| 31 | C22:0 | Behenic acid | Docosanoic acid |
| 32 | C23:0 | - | Tricosanoic acid |
| 33 | C24:1 | Nervonic acid | *Cis*-15-tetracosenoic acid |
| 34 | C24:0 | Lignoceric acid | Tetracosanoic acid |

**Table S11.** The formulation of the solution used in the ABE experiment is as follows:

| Solution | Formula |
| --- | --- |
| lysis buffer (LB) | pH 7.4, 150 mM NaCl, 50 mM Tris-HCl, 5 mM EDTA |
| lysis buffer A | LB＋(1.7%Triton X-100, 10 mM NEM, 2 mM PMSF, 2× PI） |
| 4SB solution | pH 7.4, 4% SDS, 50 mM Tris-HCl, 5 mM EDTA |
| 2SB solution | pH 7.4,2% SDS, 50 mM Tris-HCl, 5 mM EDTA |
| lysis buffer B | LB+(0.2%Triton X-100, 1 mM NEM, 1 mM PMSF, 1× PI) |
| lysis buffer C | LB+(0.2% Triton X-100, 1 mM PMSF,1× PI) |
| +NH_2_OH buffer | pH 7.4, 0.7 mol / L NH_2_OH, 1 mM HPDP-Biotin, 0.2% Triton X-100, 1 mM PMSF, 1× PI |
| -NH_2_OH buffer | pH 7.4, 50 mM Tris-HCl, 1 mM HPDP-Biotin, 0.2% Triton X-100, 1 mM PMSF, 1× PI |
| Low HPDP-Biotin buffer | pH 7.4, 150 mM NaCl, 50 mM Tris-HCl, 5 mM EDTA, 0.2 mM HPDP-Biotin, 0.2% Triton X-100, 1 mM PMSF, 1× PI |

PMSF: Phenylmethanesulfonyl fluoride (#G2008 Servicebio, China)

PI: protease inhibitors (#G2006 Servicebio, China)

Reference:

1. Lu Y, Yan J-S, Xia L, Qin K, Yin Q-Q, Xu H-T*, et al.* 2-Bromopalmitate targets retinoic acid receptor alpha and overcomes all-trans retinoic acid resistance of acute promyelocytic leukemia. *Haematologica* 2019, **104**(1)**:** 102-112.

2. Yang B, Zhang X, Gong H, Huang Y, Wang C, Liu H*, et al.* High stearic acid diet modulates gut microbiota and aggravates acute graft-versus-host disease. *Signal Transduct Target Ther* 2021, **6**(1)**:** 277.
