# Supplementary material for: Dietary long-chain fatty acids promote colitis by regulating palmitoylation of STAT3 through CD36-mediated endocytosis
Source: Cell Death Dis. 2024 Jan 17;15(1):60. doi: 10.1038/s41419-024-06456-5 (PMC10794235; doi:10.1038/s41419-024-06456-5)

Figure3H

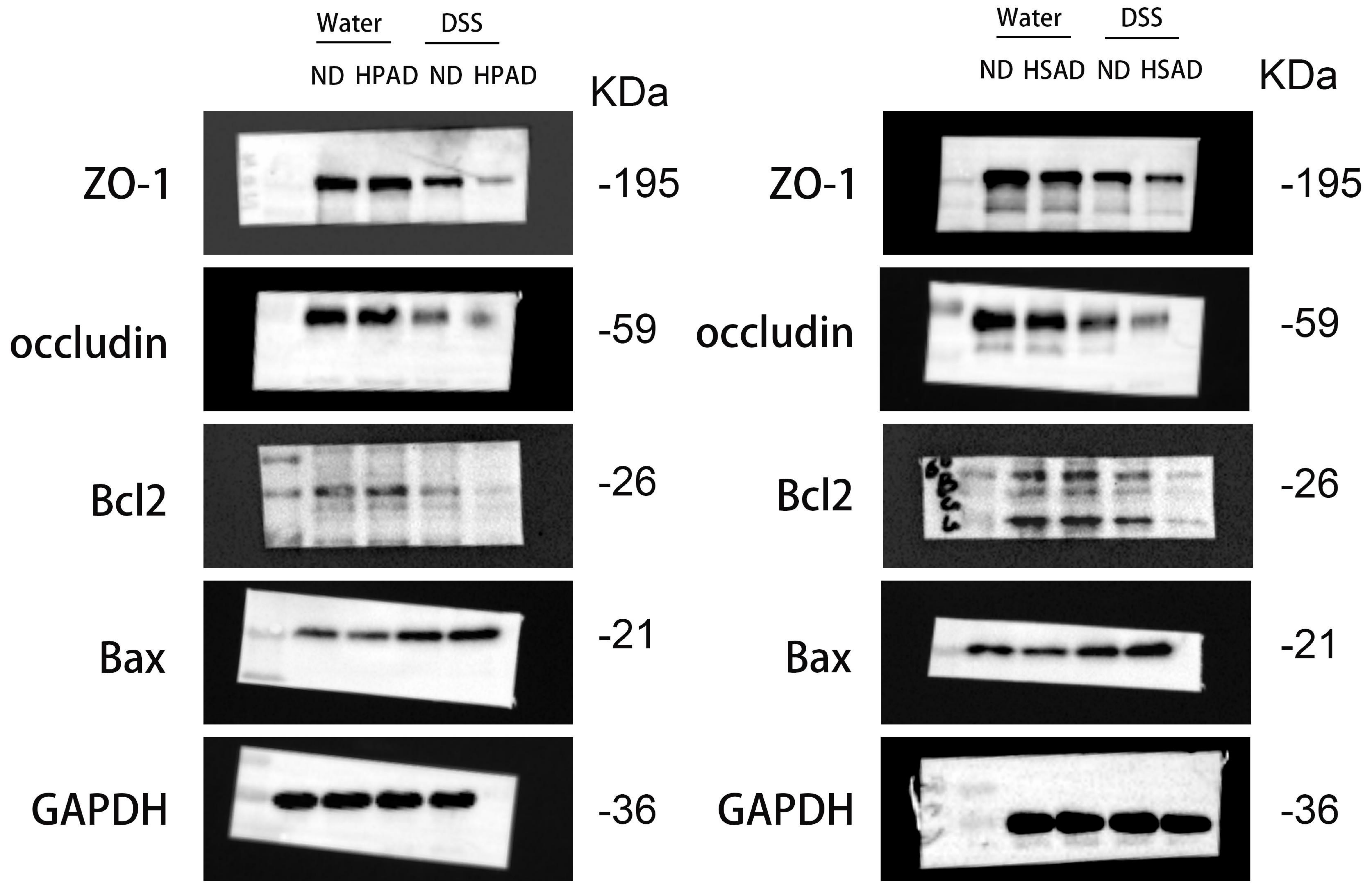

Figure 3J

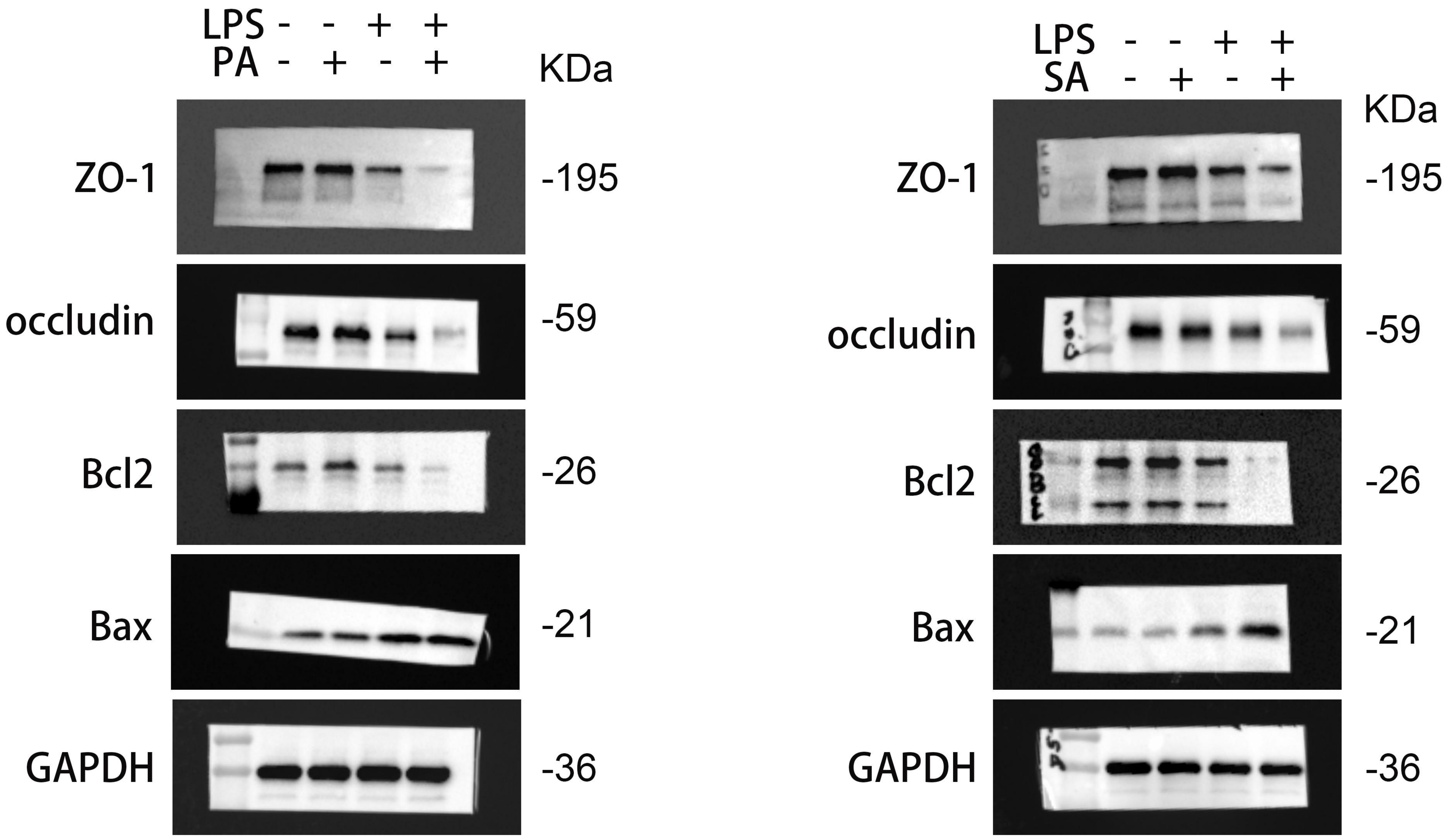

Figure 4C

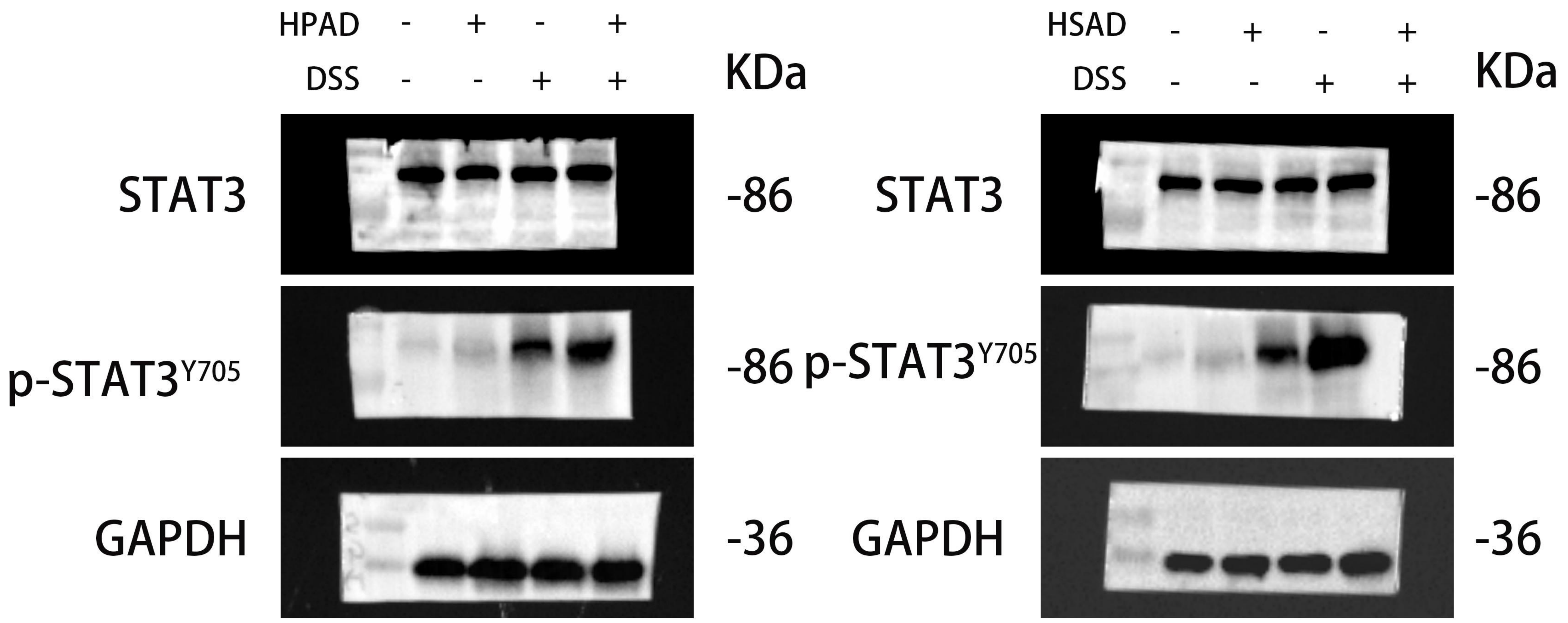

Figure 4F

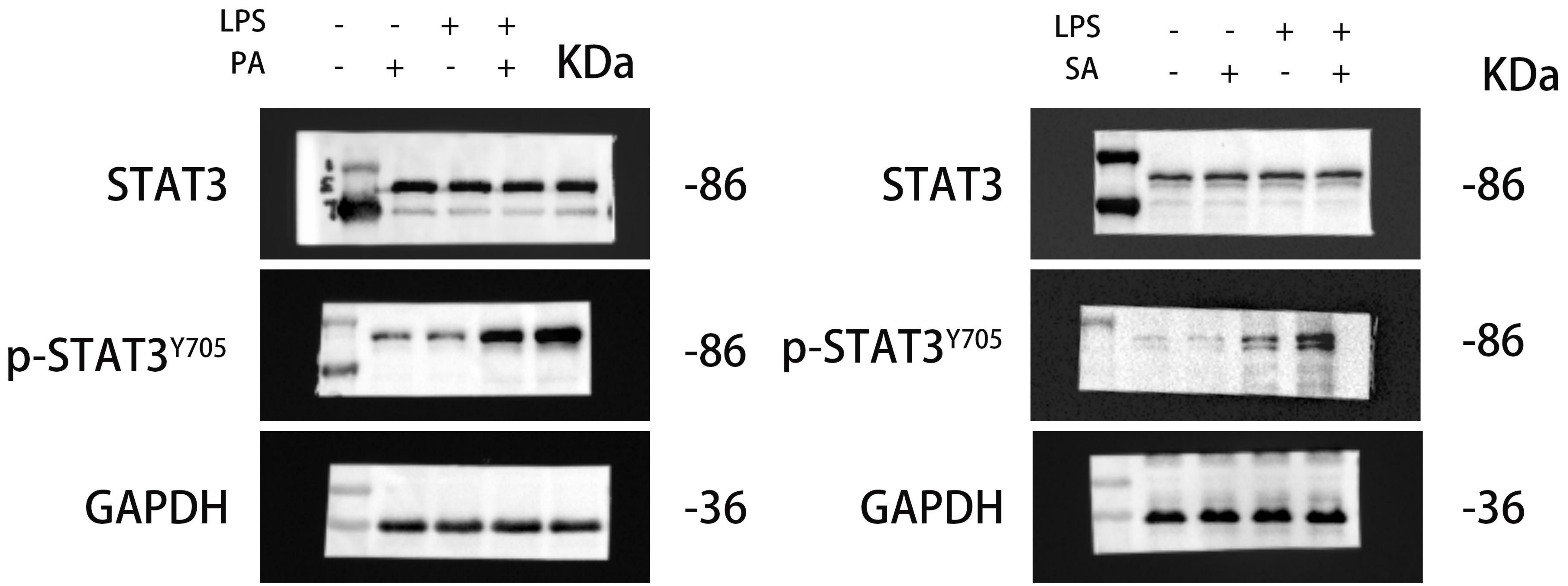

Figure 4G

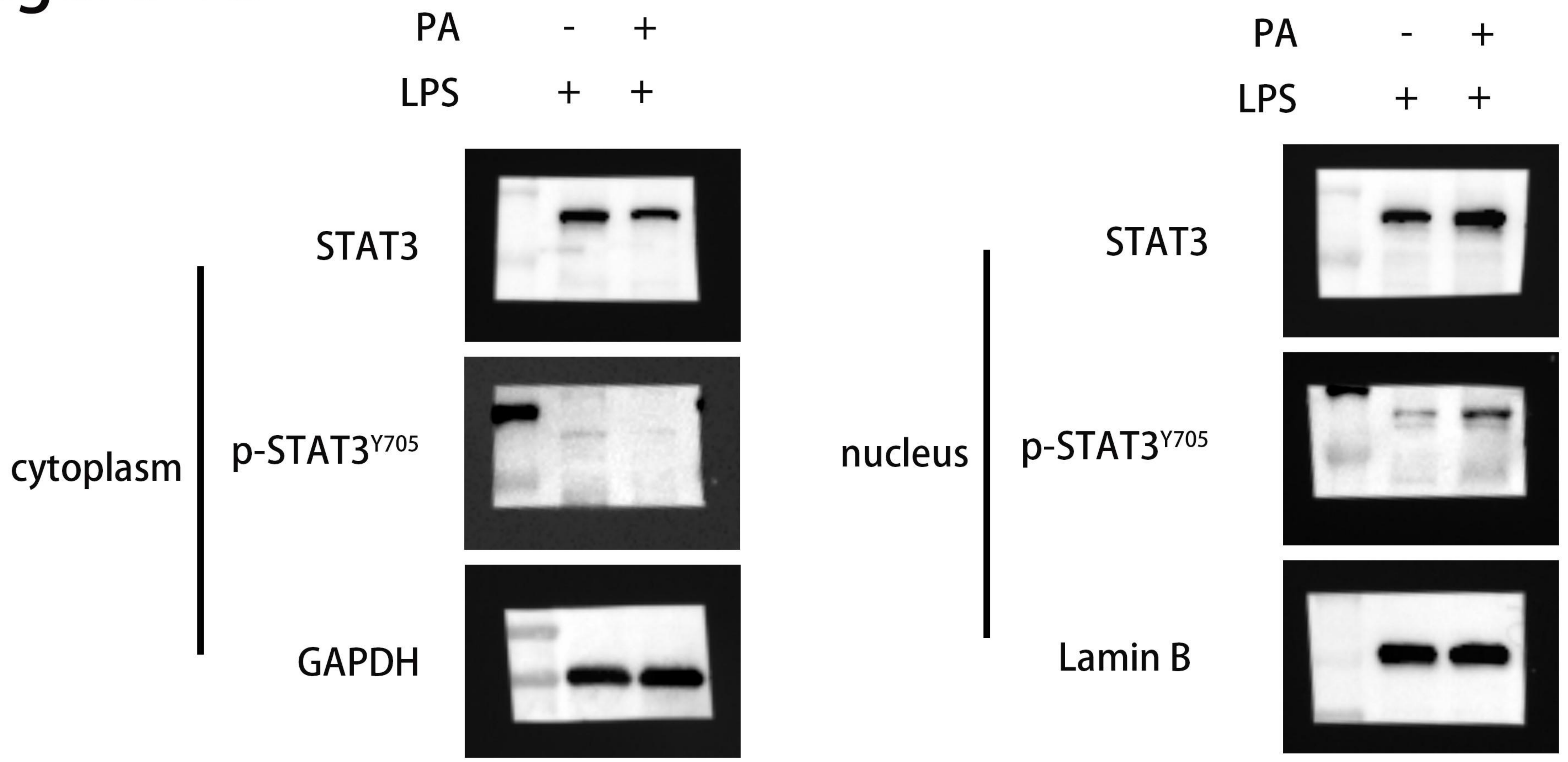

Figure 5J

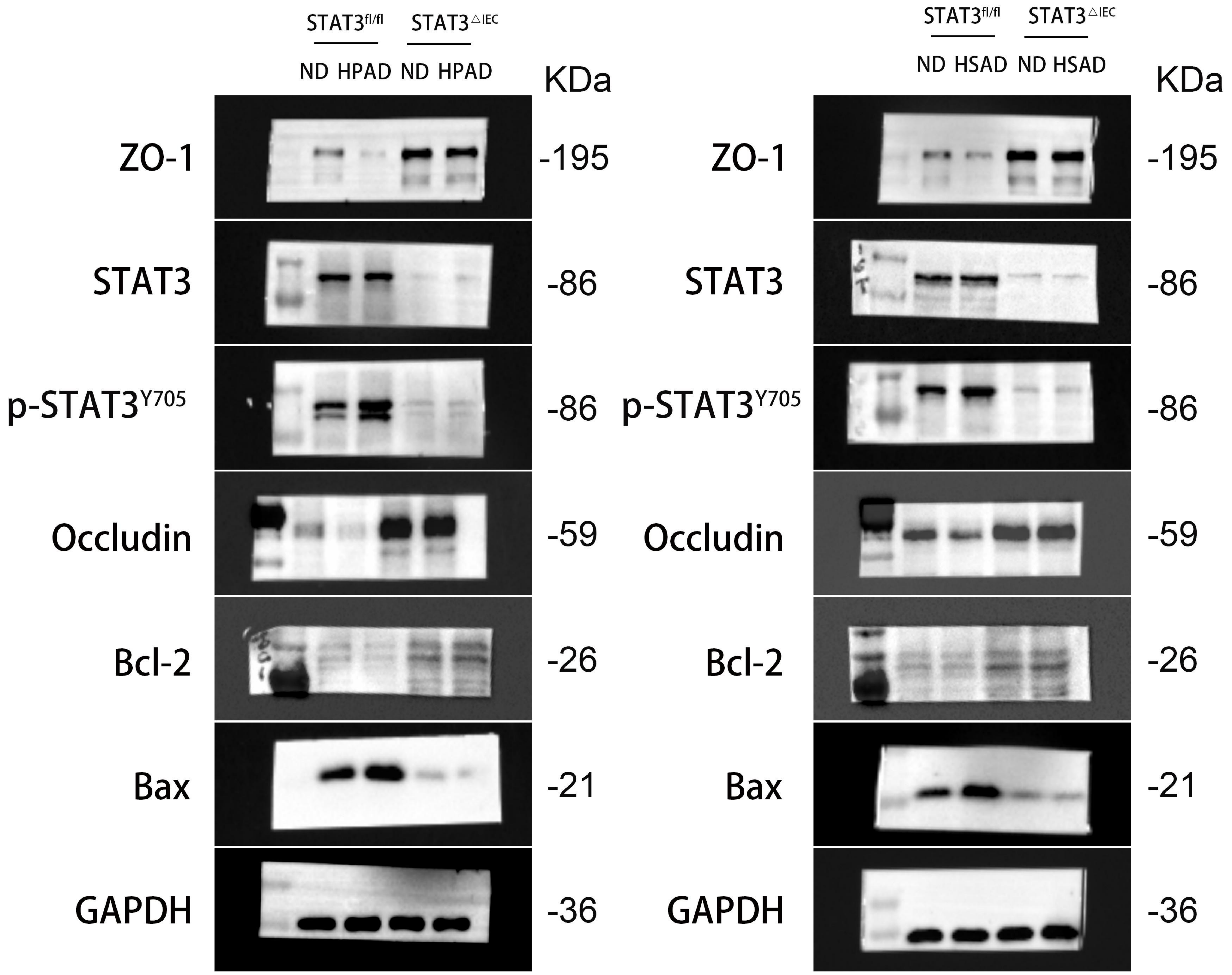

Figure 5O

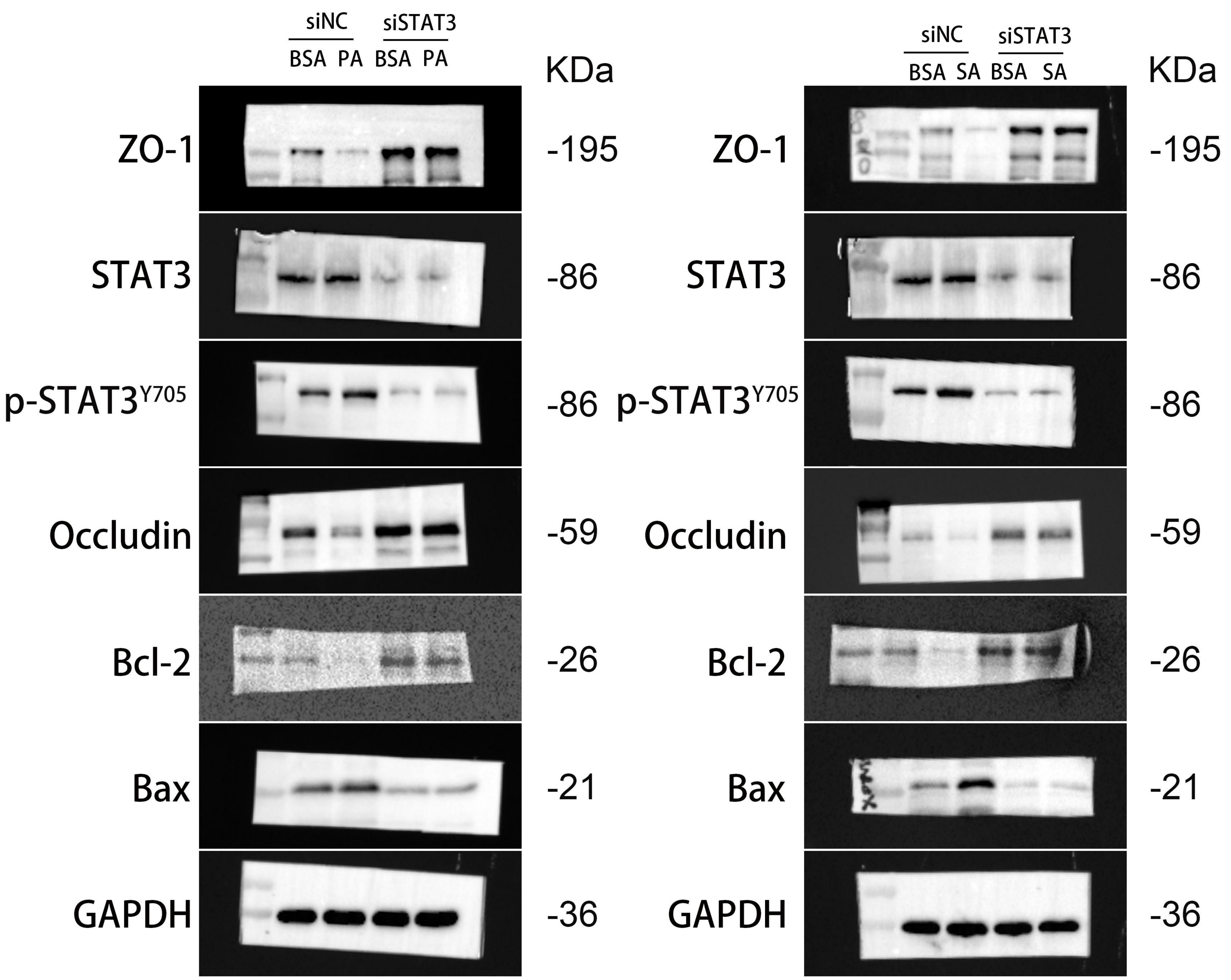

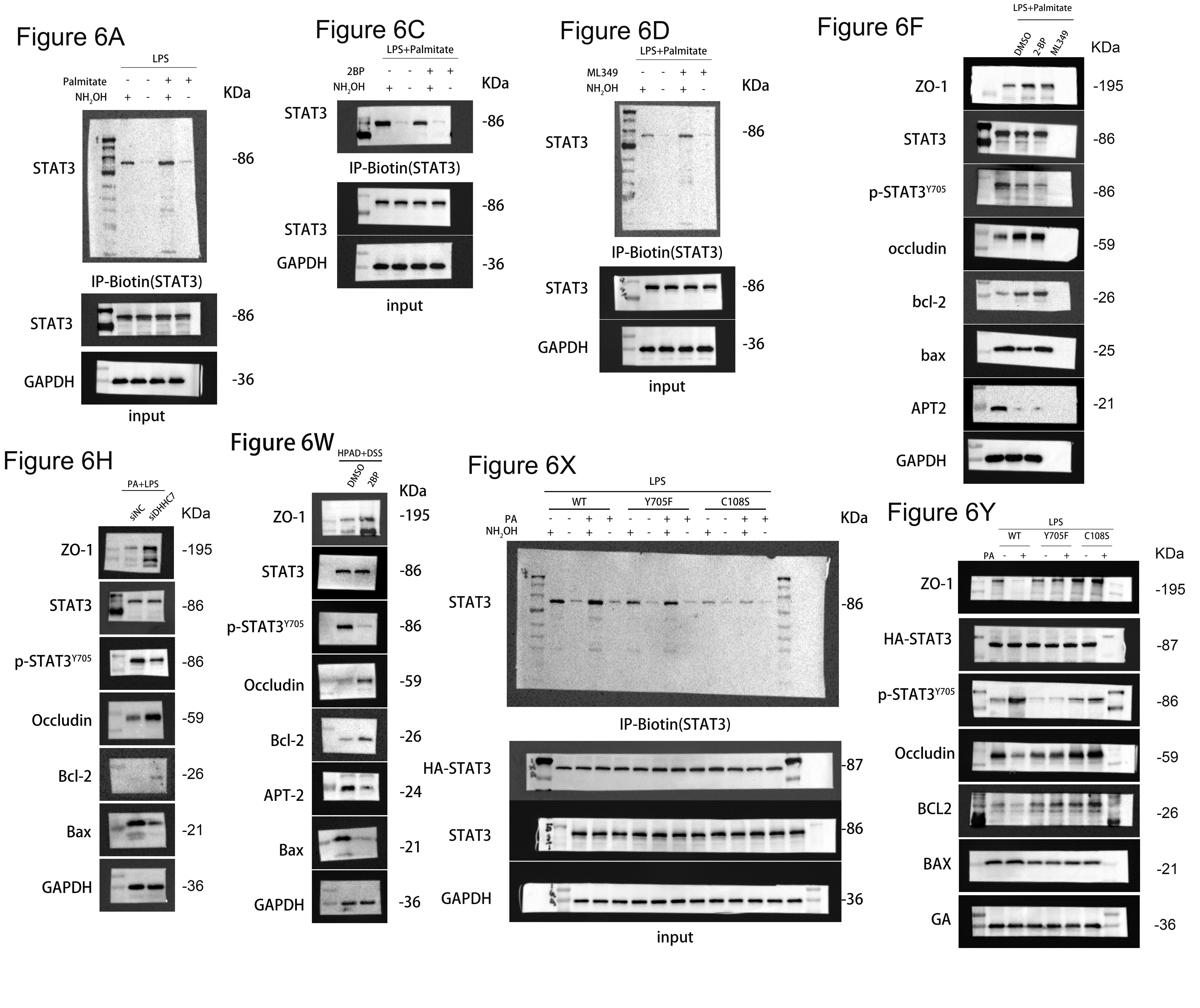
Figure 6A
LPS
Palmitate - - + +
NH2OH + - + -
KDa
STAT3 -86
IP-Biotin(STAT3)
STAT3 -86
GAPDH -36
input
Figure 6C
LPS+Palmitate
2BP - - + +
NH2OH + - + -
KDa
STAT3 -86
IP-Biotin(STAT3)
STAT3 -86
GAPDH -36
input
Figure 6D
LPS+Palmitate
ML349 - - + +
NH2OH + - + -
KDa
STAT3 -86
IP-Biotin(STAT3)
STAT3 -86
GAPDH -36
input
Figure 6F
LPS+Palmitate
DMSO
2-BP
ML349
KDa
ZO-1 -195
STAT3 -86
p-STAT3Y705 -86
occludin -59
bcl-2 -26
bax -25
APT2 -21
GAPDH
Figure 6H
PA+LPS
siNC
siDHHC7
KDa
ZO-1 -195
STAT3 -86
p-STAT3Y705 -86
Occludin -59
Bcl-2 -26
Bax -21
GAPDH -36
Figure 6W
HPAD+DSS
DMSO
2BP
KDa
ZO-1 -195
STAT3 -86
p-STAT3Y705 -86
Occludin -59
Bcl-2 -26
APT-2 -24
Bax -21
GAPDH -36
Figure 6X
LPS
WT
Y705F
C108S
PA - - + + - - + + - - + +
NH2OH + - + - + - + - + - + -
KDa
STAT3 -86
IP-Biotin(STAT3)
HA-STAT3 -87
STAT3 -86
GAPDH -36
input
Figure 6Y
LPS
WT
Y705F
C108S
PA - + - + - +
KDa
ZO-1 -195
HA-STAT3 -87
p-STAT3Y705 -86
Occludin -59
BCL2 -26
BAX -21
GA -36

Figure 7B

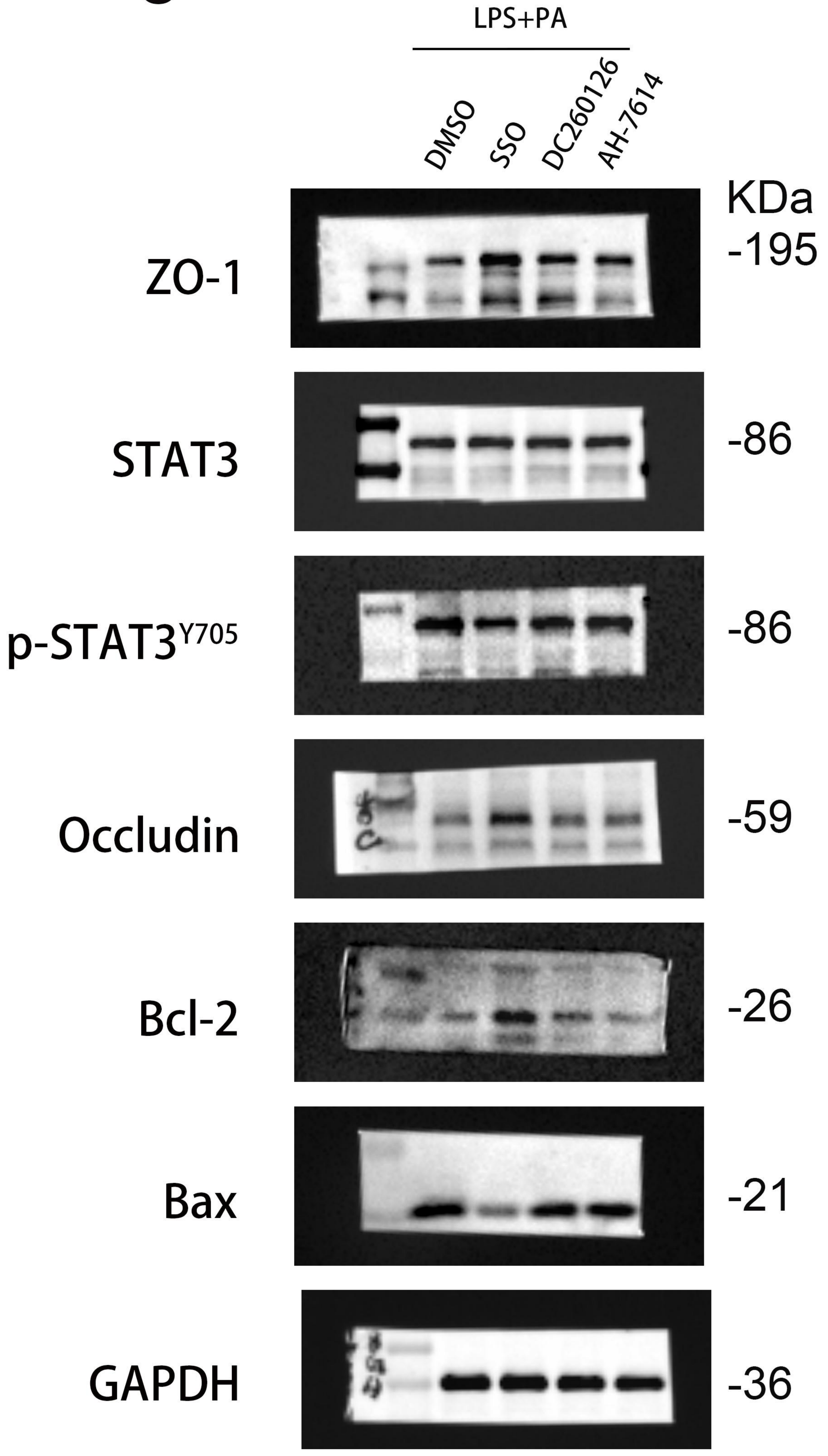

Figure 7E

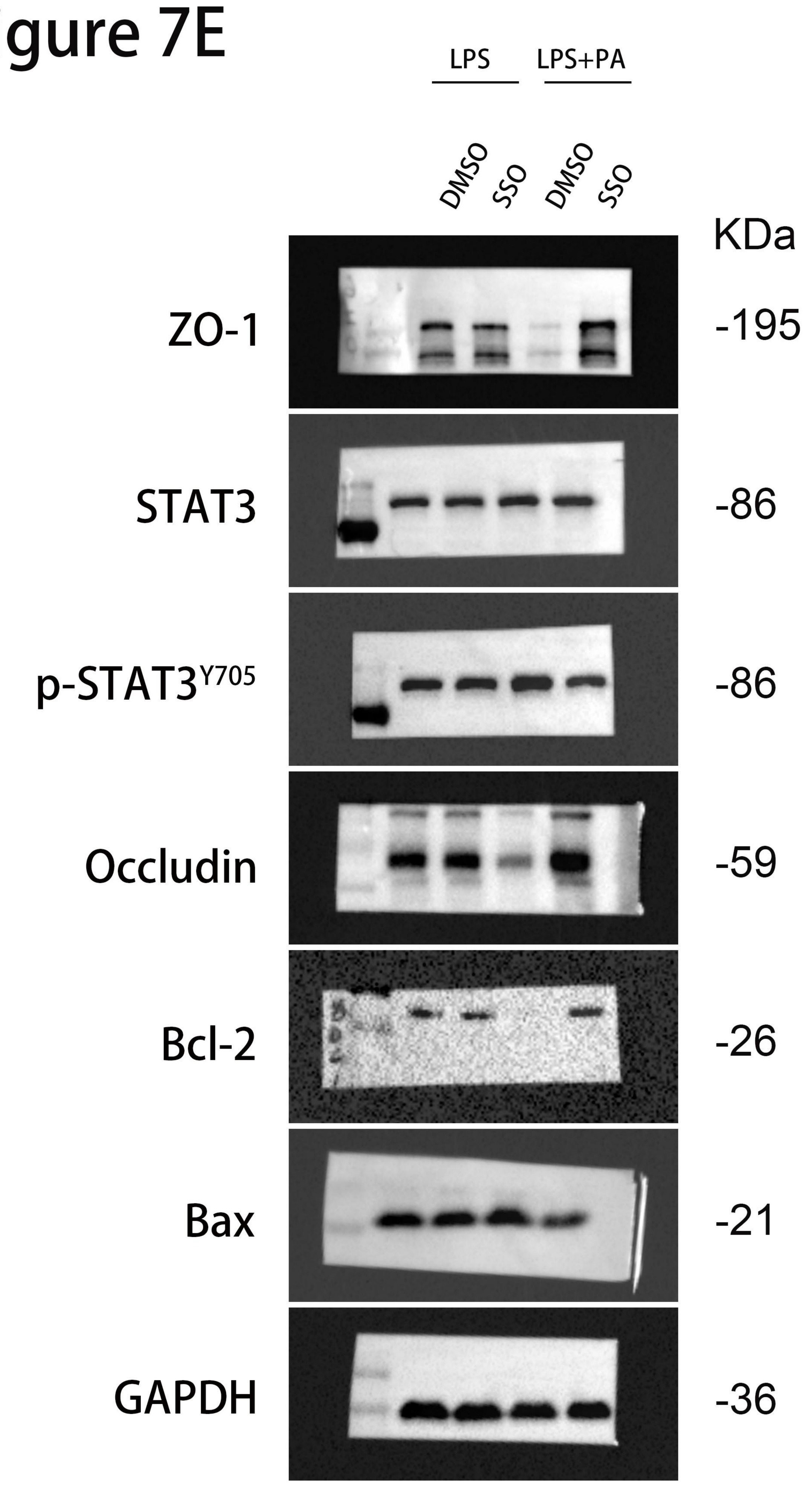

Figure 7F

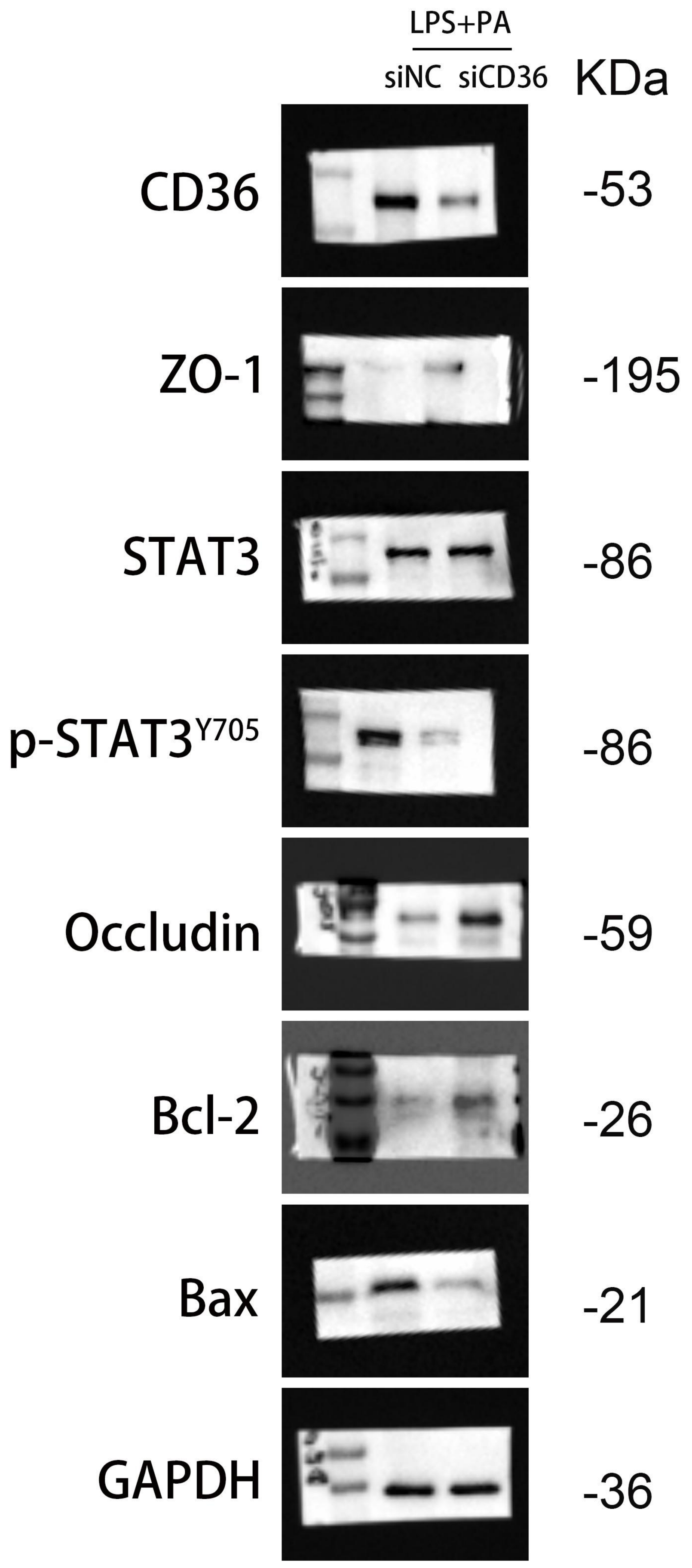

Figure 7J

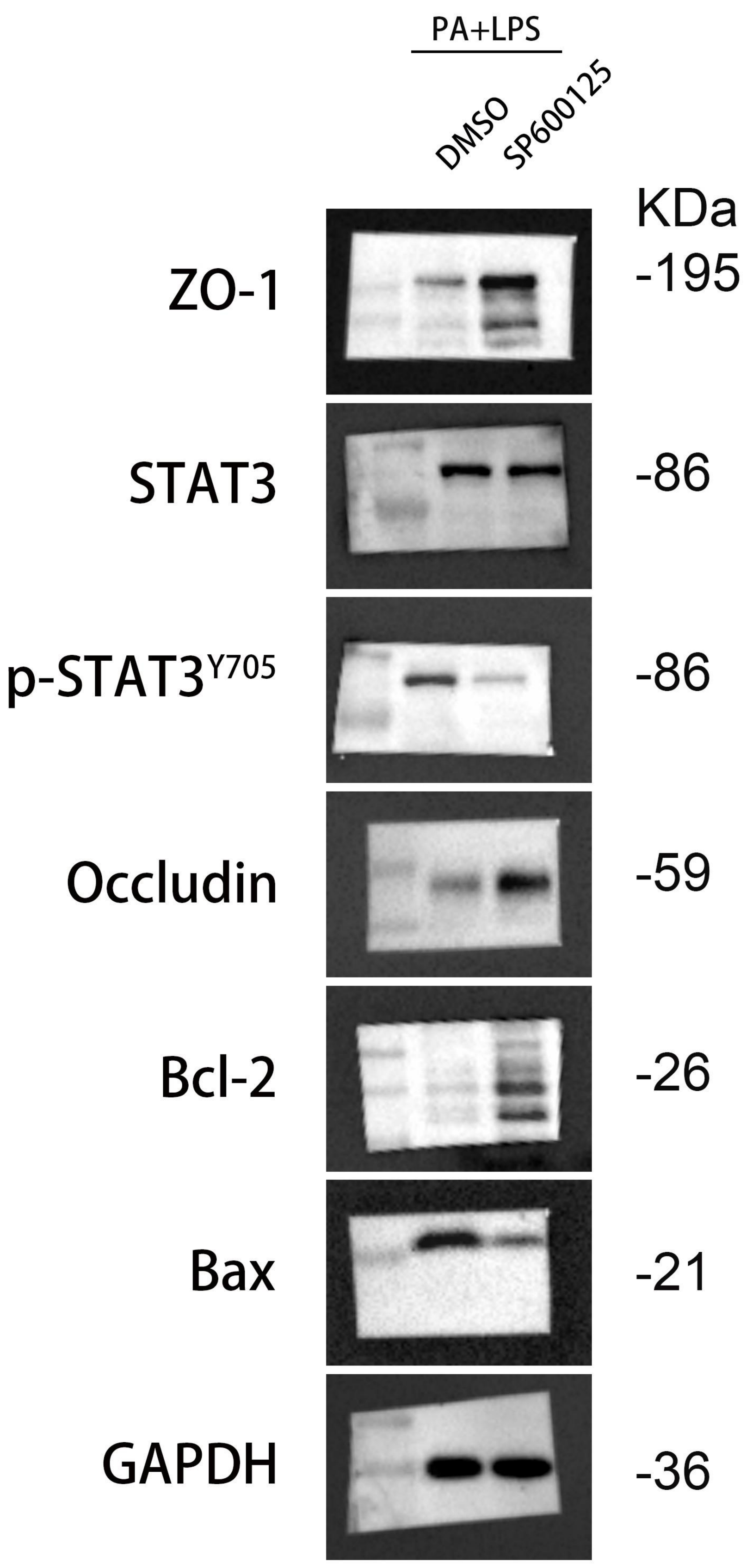

Figure 7K

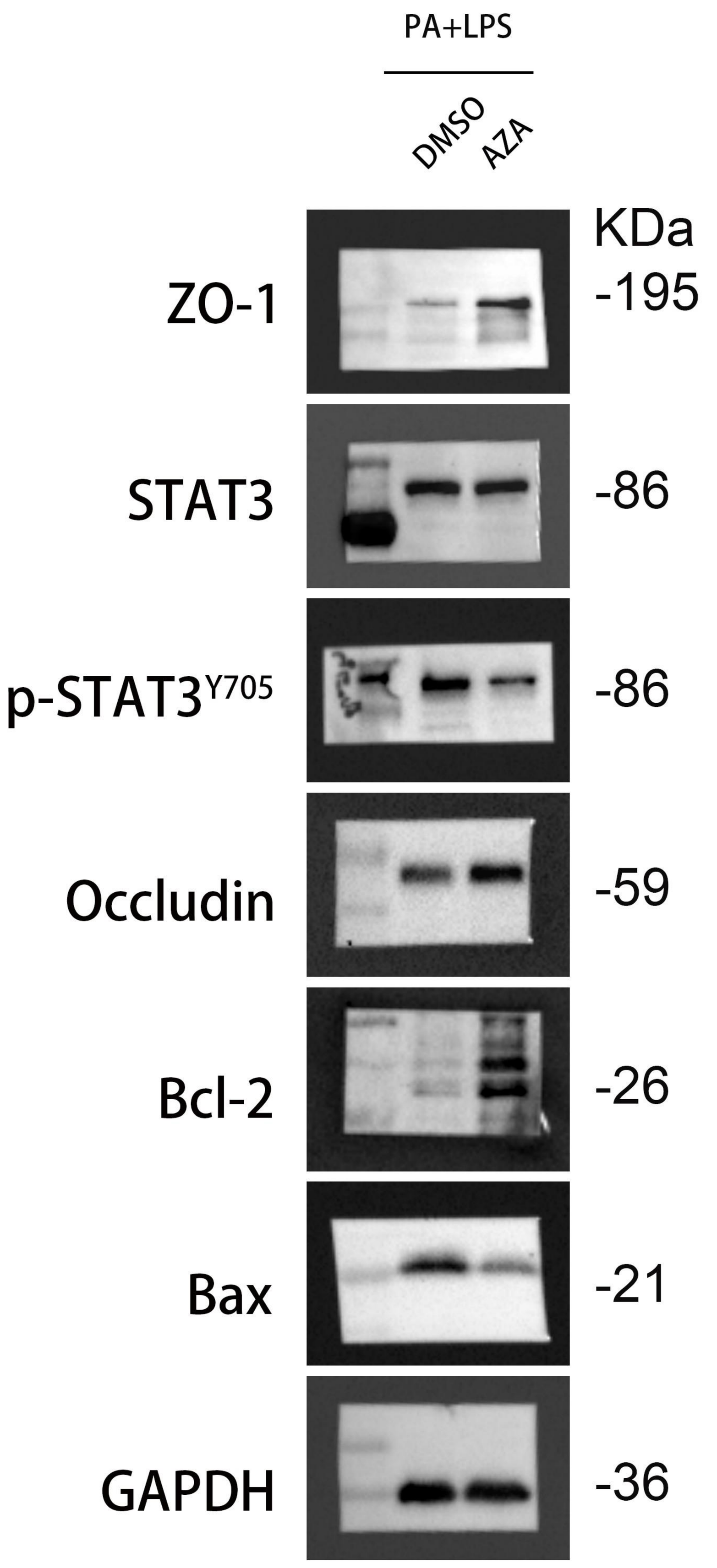

Figure 7L

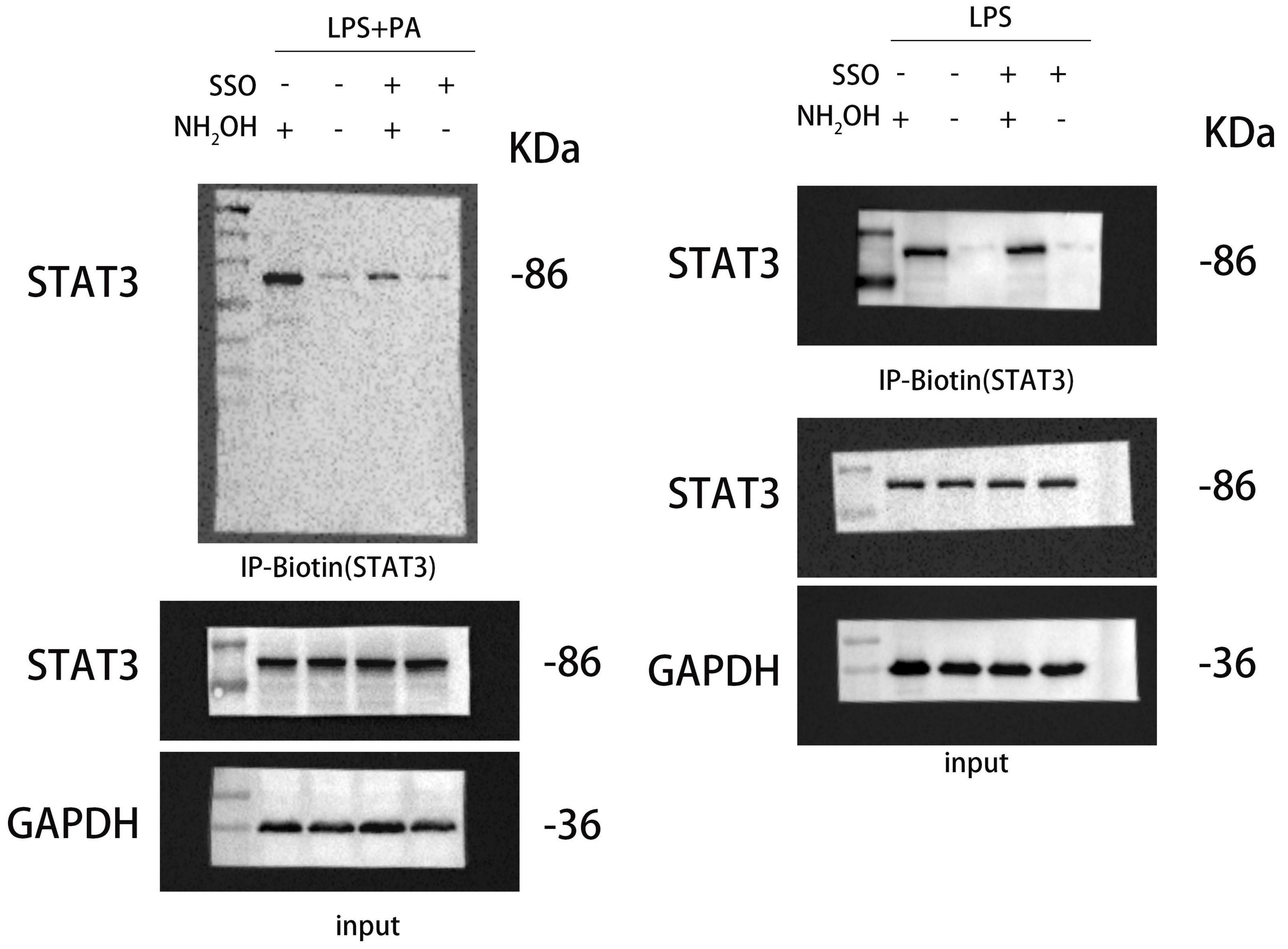

Figure S2B

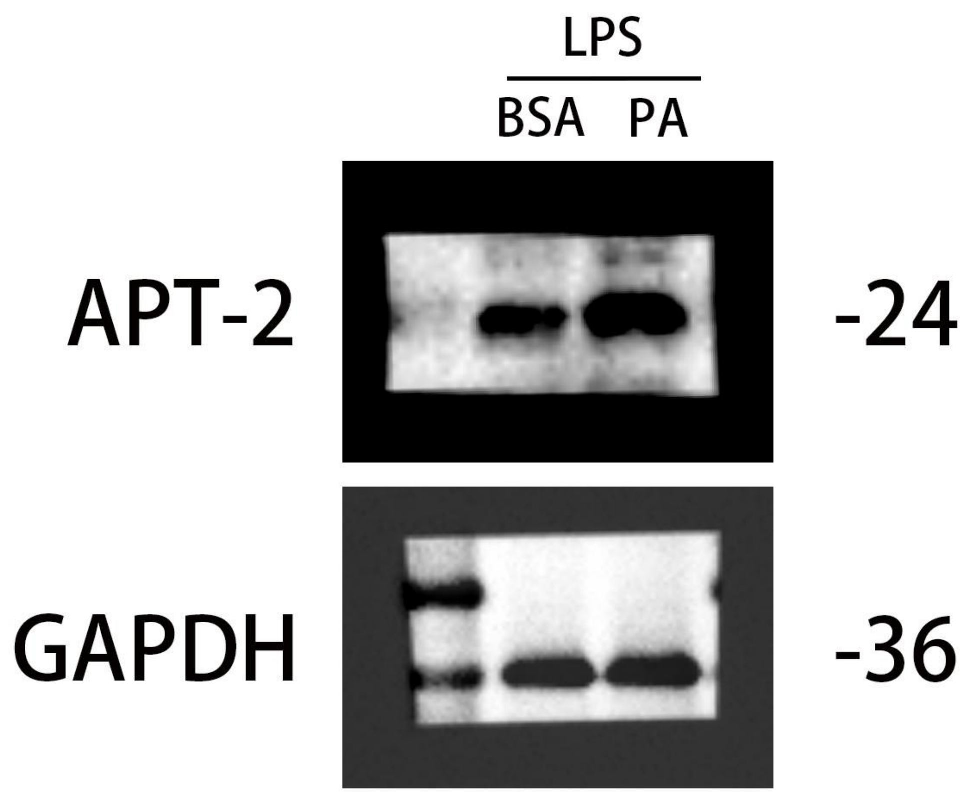

Figure S5C

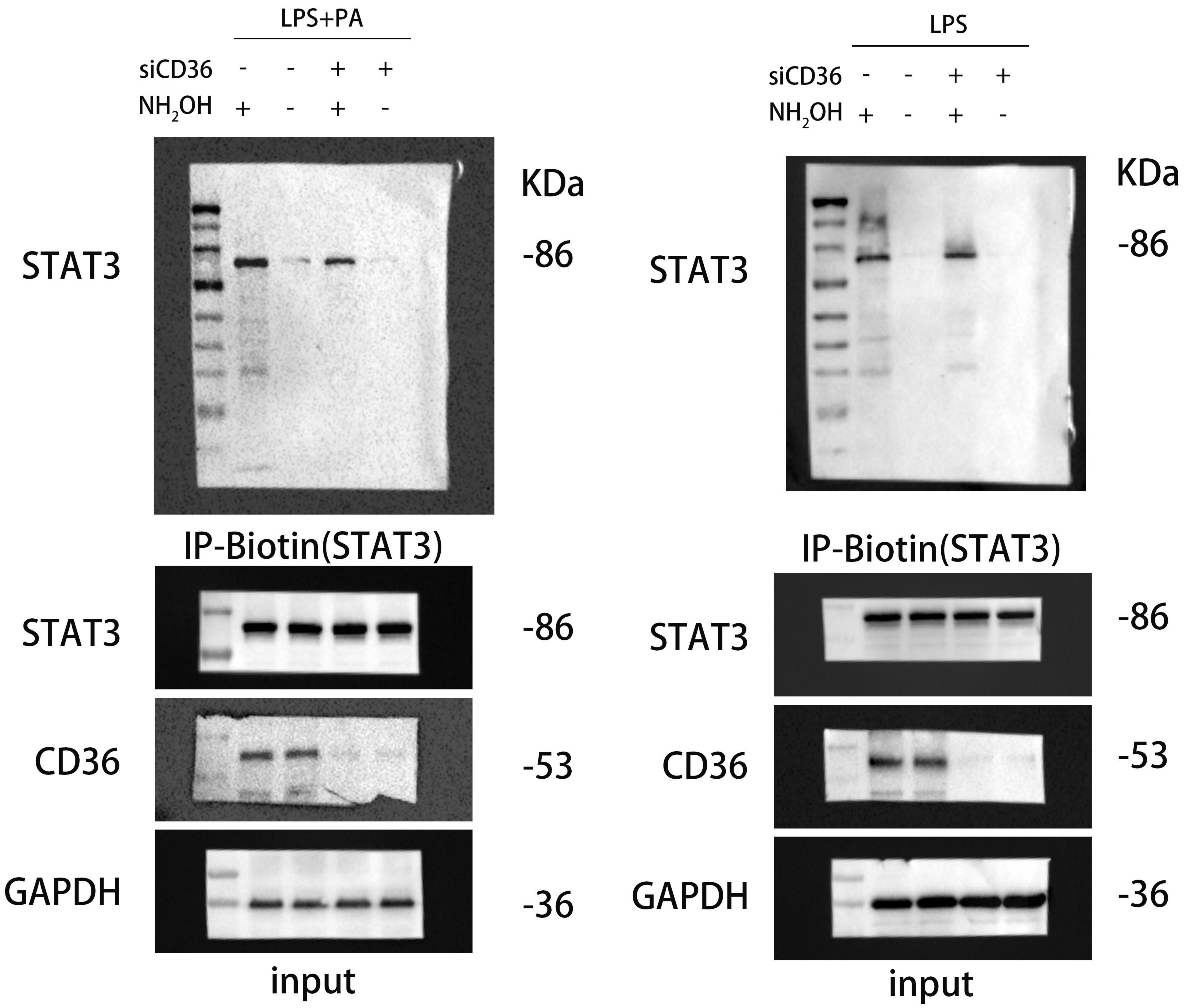

Supplement: Supplementary file 2 — Western blots [file 41419_2024_6456_MOESM2_ESM.pdf]
